# Antibacterial, Anti-Biofilm and Pro-Migratory Effects of Double Layered Hydrogels Packaged with Lactoferrin-DsiRNA-Silver Nanoparticles for Chronic Wound Therapy

**DOI:** 10.3390/pharmaceutics15030991

**Published:** 2023-03-19

**Authors:** Mohammad Aqil M. Fathil, Haliza Katas

**Affiliations:** Centre for Drug Delivery Technology, Faculty of Pharmacy, Universiti Kebangsaan Malaysia, Jalan Raja Muda Abdul Aziz, Kuala Lumpur 50300, Malaysia

**Keywords:** metal nanoparticles, biofilm, RNA interfering, biopolymer, diabetic wound

## Abstract

Antimicrobial resistance and biofilm formation in diabetic foot infections worsened during the COVID-19 pandemic, resulting in more severe infections and increased amputations. Therefore, this study aimed to develop a dressing that could effectively aid in the wound healing process and prevent bacterial infections by exerting both antibacterial and anti-biofilm effects. Silver nanoparticles (AgNPs) and lactoferrin (LTF) have been investigated as alternative antimicrobial and anti-biofilm agents, respectively, while dicer-substrate short interfering RNA (DsiRNA) has also been studied for its wound healing effect in diabetic wounds. In this study, AgNPs were complexed with LTF and DsiRNA via simple complexation before packaging in gelatin hydrogels. The formed hydrogels exhibited 1668% maximum swellability, with a 46.67 ± 10.33 µm average pore size. The hydrogels demonstrated positive antibacterial and anti-biofilm effects toward the selected Gram-positive and Gram-negative bacteria. The hydrogel containing AgLTF at 125 µg/mL was also non-cytotoxic on HaCaT cells for up to 72 h of incubation. The hydrogels containing DsiRNA and LTF demonstrated superior pro-migratory effects compared to the control group. In conclusion, the AgLTF-DsiRNA-loaded hydrogel possessed antibacterial, anti-biofilm, and pro-migratory activities. These findings provide a further understanding and knowledge on forming multipronged AgNPs consisting of DsiRNA and LTF for chronic wound therapy.

## 1. Introduction

Diabetic foot infection (DFI) is a significant concern in many parts of the world, especially in countries where diabetes is highly prevalent, such as Malaysia [1,2]. Untreated or severe DFI can result in surgical amputation of the limbs, predisposing patients to other health complications and drastically reducing their quality of life [3]. To add to the woes of diabetic patients, conventional antibiotics are becoming less and less effective due to the rise of antimicrobial resistance (AMR), leaving them with minimal pharmacological options [4,5]. Diabetic wounds also have delayed regenerative capacity due to a cascade of biological implications in high blood sugar levels, which could further aggravate the condition [6].

Increasing demand for new and alternative antibiotics to tackle AMR leads to the development of silver nanoparticles (AgNPs) as antimicrobial agents. AgNPs are heavily utilized in research settings for their antimicrobial properties and have demonstrated promising results even against resistant bacterial strains [7,8,9,10]. AgNPs offer numerous advantages as antimicrobial agents in terms of their adherence and penetration ability toward the cell surface of bacteria [11,12,13]. In addition, AgNPs that are stabilized by chitosan (CS) provide avenues for complexation with biological molecules, such as deoxyribonucleic acid (DNA) and ribonucleic acid (RNA), via their phosphorus and sulfur donor ligands [14,15].

However, with the advancement of medicines, such as humans, microorganisms, such as bacteria, have also gone through numerous evolutions to thrive in harsh and foul environments [16,17]. Bacteria can form biofilms, which are a plethora of different species living under a matrix made up of exopolysaccharides (EPS) that can protect themselves from the harmful effects of traditional antibiotics, AgNPs, and Ag^+^ due to its complex structure [18,19,20,21]. Hence, the same concentration of AgNPs usually effective against planktonic bacteria will not work the same against bacteria protected in a biofilm [11]. In addition, bacteria living in biofilm matrices can efficiently exchange nutrients and pass on plasmids containing resistance genes to promote growth and survivability [22,23,24]. Consequently, the formation of biofilms causes persistent tissue infection and can further delay wound healing in diabetic patients [25,26].

As such, the addition of lactoferrin (LTF) to AgNP therapy makes sense, as LTF can boost the subpar and strain-dependent anti-biofilm effects of AgNPs [27,28]. Basically, LTF is an iron-chelating protein found in blood and bodily secretions, such as tears, sweat, and vaginal secretions [29,30]. In reference to its anti-biofilm property, LTF has been shown to have synergistic effects in combination with agents [31], such as AgNPs [27] and Xylitol [32], against biofilm-producing organisms. Since biofilm formers are also common pathogens associated with diabetic foot ulcers [22], combining AgNPs and LTF could potentially inhibit biofilm formation and eradicate bacteria that are living in diabetic wounds.

In diabetic patients, foot ulcers are difficult to treat due to multiple factors, including defects in peripheral and endothelial vascular function [33,34]. On a cellular level, prostaglandin E_2_ (PGE_2_), which is important in maintaining proper endothelial and peripheral vascular function in the biological system, is underexpressed due to abnormally high levels of prostaglandin transporter (PGT) in hyperglycemia [35]. Dicer-substrate short interfering RNA (DsiRNA) has been extensively studied as a genetic and modern approach to silence PGT upregulation in hyperglycemic cell lines [35,36]. In vitro and in vivo studies of DsiRNA against the PGT gene have demonstrated increased VEGF levels, shortened complete wound closure time, and triple the number of blood vessels against the control [37]. DsiRNA can also form complex metallic nanoparticles with the aid of a CS monolayer due to the anionic nature of RNA [38]. This complexation of DsiRNA onto CS has been shown to improve the localization of DsiRNA in cells [39]. Hence, combining DsiRNA with LTF and AgNPs could potentially provide better healing effects due to its triple action of healing, antibacterial, and anti-biofilm properties. 

Hydrogels are commonly used as a drug delivery system for wound healing, as they are biocompatible, have a high water content, and have a rubbery consistency [40,41,42]. In addition, hydrogels provide moist and suitable environments conducive to wound re-epithelialization of tissues [43]. They can also utilize nanoparticles as a drug depot and significantly prolong the drug release duration, which increases drug bioavailability and patient compliance [44,45]. In this instance, gelatin is often used as a base for hydrogels because it is cheap, easily accessible, has excellent cell adhesion properties, low antigenicity, and versatile chemical and physical modifiability [46,47]. However, the downside of gelatin is that it has unstable mechanical properties under physiological conditions, despite the presence of physically crosslinked nanoparticles. This necessitates using chemical crosslinkers to enhance and strengthen connective chains from faltering in the medium [48,49]. Employment of both physical and chemical crosslinkers in a hydrogel could permanently fix the shape at rest, showing low fracture toughness and extensibility. This is ideal in a hydrogel that requires the extended release of active components [50]. Such characteristics are important because sequential and prolonged-release therapy, according to the wound healing stages that consist of the initial inflammation stage followed by a proliferation stage of up to 10 days, has been shown to have an enhanced healing effect [51]. 

Developing multifunctional hydrogel dressings that are able to eliminate bacterial infections and accelerate the diabetic wound healing process is often desirable to address these issues associated with diabetic wounds [43,52]. In the present study, synthesized AgNPs complexed with LTF and DsiRNA are separately compartmentalized in a double-layered hydrogel to produce a three-way therapy to combat biofilm-producing pathogens and, at the same time, expedite the healing capacity of human skin cells, which are released in accordance with the wound healing stages. The proposed treatment offers advantages, including being easy to produce, as it does not involve lengthy protocols and sophisticated instruments. Furthermore, it is also environmentally friendly, as it promotes using natural compounds to synthesize AgNPs. Both the nanocomposites and the gel were characterized for physical characteristics and drug release behaviors. The antibacterial and anti-biofilm properties of AgNPs and LTF were determined using a microbroth dilution test and crystal violet assay, whereas the in vitro migration quantification of cells after treatment with AgNP-DsiRNA was determined via the wound scratch assay. The gels that demonstrated enhanced healing properties while effectively eradicating common biofilm-forming pathogens would be very effective for application as a therapy for diabetic wounds.

## 2. Materials and Methods

### 2.1. Materials

TMM powder (Lignosus rhinocerotis) was received from Lignas Bio Synergy Plt., Sepang, Malaysia, as a donation. Silver nitrate (AgNO_3_) (ACS reagent grade), low molecular weight (LMW) CS (50–190 kDa, 75–85% deacetylated), and human recombinant LTF (expressed in rice, iron-saturated and >90% SDS PAGE) were procured from Sigma Aldrich, MO, USA. Ciprofloxacin HCl was obtained from TargetMol, MA, USA, whereas Ciprofloxacin in 5 µg disks was purchased from Thermo Fisher Scientific, MA, USA. Three bacterial strains (Staphylococcus aureus ATCC 25923, Pseudomonas aeruginosa ATCC 27853, and Escherichia coli ATCC 25927) were requested and received from Universiti Kebangsaan Malaysia, Kuala Lumpur, Malaysia. Mueller-Hinton broth (MHB) and Mueller-Hinton agar (MHA) were obtained from TargetMol, MA, USA. Crystal violet was purchased from Chemiz, Shah Alam, Malaysia. Ethanol (70%) was procured from J-Kollin Chemicals, Midlothian, UK, and glacial acetic acid (99.7% purity) was purchased from R&M Chemicals, Subang, Malaysia. Both gelatin and genipin, which were used to form hydrogel bases, were procured from Sigma Aldrich, MO, USA. 

A human epidermal keratinocyte cell line (HaCaT) (passage 0) was purchased from Addexbio, CA, USA, for cell viability and migration tests. DMEM high glucose (4.5 g/L D-(+)-Glucose) and low glucose (1.0 g/L D-(+)-Glucose) with pre-added supplements L-Glutamine, phenol red, and sodium pyruvate were procured from Nacalai Tesque, Kyoto, Japan. AlamarBlue (AB) cell viability reagent was procured from Invitrogen, MA, USA. Pen-Strep (penicillin/streptomycin) and fetal bovine serum (FBS) were purchased from Tico Europe, Amstelveen, Netherlands. Protein extraction from the cells was done using radioimmunoprecipitation assay (RIPA) buffer, procured from Sigma Aldrich, MO, USA, and phenylmethylsulfonyl fluoride (PMSF) as a protease inhibitor, which was purchased from Roche, Basel, Switzerland. Prior to the assay, 1 M PMSF stock solution was prepared by dissolving 1.742 g of PMSF powder in 10 mL of dimethyl sulfoxide (DMSO) and stored at −20 °C. 

### 2.2. Preparation of Biosynthesized AgNPs and Functionalization with LTF

AgNPs were synthesized according to a previously reported method [53]. Briefly, 5 mL of water extract of tiger milk mushroom (WETMM) (0.1 mg/mL) was mixed with 1 mL of 0.01 M silver nitrate solution (0.017% *w*/*v*) and 5 mL of CS solution (0.09% *w*/*v*). The reaction was performed at room temperature (RT) and left to stand until the yellow color turned reddish brown (the target color), indicating the formation of AgNPs. The mixture was sonicated for 20 min, followed by stirring at 300 rpm for 30 min. The resulting mixture was washed three times with deionized water by centrifugation (15,000 rotations per minute (rpm) for 15 min) to remove excess unreacted reactants. Formed AgNPs were re-suspended in 5 mL of distilled water and incubated with 5 mL of LTF solution to produce AgLTF complex at various concentrations (Ag/LTF concentration: 1000/2000, 500/1000, 250/500, 125/250, and 62.5/125 µg/mL).

The formation of the AgLTF complex was analyzed using a UV-vis spectrophotometer (Shimadzu 180, Centurion Scientific, New Delhi, India). The scan range was 200–500 nm at a 480 nm/min scan speed. Biosynthesized AgLTFs were kept at −80 °C for three days before lyophilization. Then, AgLTF was freeze-dried in a freeze dryer (ScanVac CoolSafe, Labogene, Lillerød, Denmark) at −110 °C for 24 h, and the lyophilized samples were viewed under a transmission electron microscope (TEM) at different magnifications (Philips, CM12, CA, USA).

### 2.3. FTIR Analysis

Samples for Fourier transform infrared spectroscopy-attenuated total reflectance (FTIR-ATR) were prepared by suspending lyophilized AgLTF and LTF in distilled water. Then, a few drops of the samples were placed on the ATR crystal. FTIR-ATR analysis of AgLTF and LTF was conducted in the range of 4000–400 cm^−1^ using an FTIR spectrophotometer (Perkin Elmer 100 Spectrum, Walthman, MA, USA). The spectra were acquired using 32 scans and a 4 cm^−1^ resolution.

### 2.4. X-ray Diffraction Analysis

X-ray diffraction (XRD) patterns were recorded using a D8 Advance X-ray diffractometer (Bruker, Rheinstetten, Germany) equipped with a high-speed energy-dispersive LYNXEYE XE-T detector to characterize the structure and crystallinity of LTF, AgNPs, and AgLTF. Before analysis, the samples were prepared via lyophilization and kept in an airtight container.

### 2.5. DsiRNA Adsorption to AgLTF

Similarly, 1 mL of various concentrations of AgLTF suspensions (125 µg/mL) was added to 1 mL DsiRNA against PGT gene solution (0.015 µg/mL), and the mixture was incubated at RT for 30 min to produce AgLTF-DsiRNA.

### 2.6. Particle Size, Polydispersity Index (PDI), and Zeta Potential

The mean particle size, polydispersity index (PDI), and zeta potential (surface charge) of prepared biosynthesized AgNPs, AgLTF, and AgLTF-DsiRNA were determined using a Malvern Zetasizer Nano ZS (Malvern Instruments, Worcestershire, UK). All measurements for particle size were performed at 25 °C with a detection angle of 90°. Samples were measured in triplicate, and the data were presented as the mean ± standard deviation.

### 2.7. DsiRNA Entrapment and Binding Efficiency

The entrapment efficiency (EE) of DsiRNA complexed with AgLTF was measured using a UV-vis spectrophotometer. The samples were centrifuged at 10,000 rpm for 30 min, and the absorbance of the supernatant recovered from centrifugation was measured at 260 nm and scanned at 480 mm/min. The following Equation (1) was used to calculate DsiRNA EE [39]:

(1) $$\mathrm{DsiRNA}\;\mathrm{EE}\;\%=\frac{\mathrm{DsiRNA}\;\mathrm{added}\;\mathrm{OD}260\;\mathrm{nm}-\mathrm{DsiRNA}\;\mathrm{in}\;\mathrm{supernatant}\;\mathrm{OD}260\;\mathrm{nm}}{\mathrm{DsiRNA}\;\mathrm{added}\;\mathrm{OD}260\;\mathrm{nm}}\;\cdot \;100$$

The binding efficiency (BE) test was conducted using the electrophoresis method. Agarose gel 5% was prepared in a 60 mL TAE buffer with 2 µL of red gel stain (Vivantis, Shah Alam, Malaysia). Samples were prepared by mixing dye with samples at a 1:5 ratio. Samples (10 µL) containing different concentrations of AgLTF with DsiRNA were then loaded into the wells prior to electrophoresis. Each sample contained 56 ng of RNA. Naked DsiRNA was used as the positive control, blank gel as the negative control, and a 10 bp DNA ladder (Invitrogen, MA, USA) as a size reference. The gel was run at 70 V for 45 min. The migration of the RNA in the agarose gel was captured and viewed under the ChemiDoc XRS+ System (Bio-Rad Laboratories, Hercules, CA, USA) using Image Lab software.

### 2.8. Preparation of the Hydrogel

Two kinds of hydrogels were prepared in this study: a simple single-layered (SL) hydrogel and a double-layered (DL) hydrogel consisting of different complexes (AgNPs-DsiRNA and AgLTF) in each layer. DL hydrogels were proposed as a means of modulation and prolonged release of DsiRNA in the upper layer. The hydrogel was prepared using a method and formula reported previously with slight modifications [54].

Briefly, in a DL hydrogel, 5.7 mL AgLTF suspensions were mixed with 180 mg of gelatin to make 3% *w*/*v* gelatin as a hydrogel base and were left on a hot plate at 40 °C for 30 min until the powders were fully dissolved [54]. Then, 0.3 mL genipin solution (0.005% *w*/*v*) was added to the mixture to make a total volume of 6 mL of gel. The cross-linking process was left for 24 h at RT until the gel was fully manifested to form the first or lower layer of DL hydrogel. Similarly, pre-incubated AgNPs-DsiRNA was mixed with gelatin (3% *w*/*v*) and genipin of various concentrations (0.005, 0.01, 0.02, and 0.04% *w*/*v*) to make up a 6 mL volume of the hydrogel. After fully dissolved gelatin powder, the mixture was poured onto the first layer of gel to form the second or upper layer of the DL hydrogel.

As for SL hydrogel, 5.7 mL pre-incubated AgLTF-DsiRNA was mixed with gelatin (3% *w*/*v*) and genipin (0.005% *w*/*v*) at 40 °C for 30 min until the gelatin powder was dissolved fully. The mixture was left for 24 h for the cross-linking process to complete. Then, the formed gels’ visual appearance, texture, smoothness, and stickiness were evaluated. DL and SL gels were also inverted and inclined for 1 h to observe their adhesion.

### 2.9. Morphological Observation

Scanning electron microscope (SEM) imaging of hydrogels was taken using Merlin at magnification 500–1000× (Zeiss, Wetzlar, Germany) to observe 3-dimensional (3D) microporous structures of the hydrogels. The prepared hydrogels were kept at −80 °C in a freezer for 3 days and were lyophilized at −110 °C for 24 h using a freeze dryer before analysis.

### 2.10. Swelling Capacity

The swelling behavior of the hydrogels was studied to determine their maximum hydration capacity. First, the hydrogels were freeze-dried for 24 h prior to the test. The swelling capacity (I_s_, %) of gelatin hydrogels was measured by weighing approximately 100 mg of dried sample (W_d_) followed by immersion in 50 mL of phosphate buffer saline (PBS) with pH 7.4 at RT. The hydrogels were then superficially dried using filter paper, and the wet weight (W_w_) was taken every 30 min up to 5 h and at 24 h. I_s_ % was calculated using Equation (2) below [55]:

(2) $$I_{s}\;\%=\frac{\mathrm{Ww}-\mathrm{Wd}}{\mathrm{Wd}}\;.\;100$$

### 2.11. In Vitro Drug Release of Active Agents

In vitro release of AgNPs, LTF, and DsiRNA from the hydrogels was determined using Franz diffusion cells (PermeGear Inc, Hellertown, PA, USA). Cellulose acetate (Sartorius Stedim Biotech, Göttingen, Germany) with a pore size of 0.45 µm was used as the membrane, which was fixed between the donor and the receptor chamber. The diffusion area of the orifice between the donor and receptor chamber was 0.7855 cm^2^. The receptor chamber was filled with 5 mL PBS (pH 7.4 and 8.0) and maintained at 37 ± 2 °C throughout the process. A magnetic stirring bar was also added to the receptor chamber containing PBS to preserve the homogeneity of the system. Hydrogel samples (1 mL) were then directly inserted into the donor chamber and allowed to flow until the gels settled on the membrane. As for DL hydrogels, they were directly placed on the membrane without the donor chamber to maintain their integrity and shape to accurately represent the release profile of active agents from the respective upper and lower layers. Sampling was done by drawing 0.5 mL of PBS in the receptor chamber via a sampling port at predetermined time points (1, 2, 3, 4, 5, 6, 7, 8, 24, 48, and 72 h). Then, 0.5 mL of the drawn sample was immediately replenished with the same volume of fresh PBS. The absorbance of the samples was analyzed using a UV-vis spectrophotometer at 260 nm for DsiRNA, 280 nm for LTF, and 430 nm for AgNPs. Each active component’s percentage drug release (%) was plotted against time (h). As for DsiRNA, the percentage of drug release (%) was adjusted based on the average EE calculated.

### 2.12. Antibacterial Assays

#### 2.12.1. Inoculum Preparation by Growth Method

*S. aureus* (ATCC 25923), *E. coli* (ATCC 25927), and *P. aeruginosa* (ATCC 27853) were cultured on agar plates containing MHA using the streak method and then incubated (Memmert, Büchenbach, Germany) for 18 h at 37 °C. Inocula were prepared by transferring 3–5 colonies into a universal bottle containing 5 mL of MHB using a sterile loop. The bacterial suspension was incubated overnight to allow bacterial growth. After 18 h, the turbidity was adjusted using a UV-vis spectrophotometer to an absorbance of 0.08–0.10 at 625 nm by adding sterile broth to obtain a standardized microbial suspension of 1 × 10^8^ CFU/mL for all bacterial strains.

#### 2.12.2. Microbroth Dilution Method

The minimum inhibitory concentration (MIC) value of AgLTF against the selected pathogens was determined using the microbroth dilution method [56,57]. Serial dilutions were performed from a starting concentration of 1000 to 2 µg/mL using MHB as a diluent. Each dilution reduced the concentration by two-fold. Then, a 1 × 10^8^ CFU/mL bacterial suspension was prepared by adjusting the overnight (18 h) bacterial cultures using a UV-vis spectrophotometer. The bacterial suspension was then diluted with MHB at a 1:100 ratio to obtain approximately 1 × 10^6^ CFU/mL. Subsequently, 100 µL of each type of bacterial strain was dispensed into each well of a 96-well plate containing 100 µL of treatment samples to give a final bacterial concentration of 5 × 10^5^ CFU/mL. Finally, the plates were incubated at 37 °C for 18 h. Then, the plates were stained using 20 µL triphenyl tetrazolium chloride (TTC) reagent (0.2% *w*/*v*), and the results were obtained by observing the presence of a red formazan color. The lowest concentration at which no visible growth (no formation of red color) occurred was noted as the MIC value of the samples.

#### 2.12.3. Agar Well Diffusion Test

The antimicrobial properties of AgLTF (1000 and 125 µg/mL) and LTF (125, 250, 500, and 1000 µg/mL) were determined using the agar well diffusion method. The test protocols of this study were taken from a method reported previously with some modifications [57]. Adjusted bacterial suspensions with a concentration of 1 × 10^8^ CFU/mL for all three bacterial strain cultures were prepared and spread on the surface of MHA by using a sterile cotton swab. The agar surface was swabbed uniformly with the bacterial suspensions by rotating several times and pressing firmly to cover the entire agar, including its rim. Four wells were created on the agar plate using a sterile 6 mm diameter pipette tip. Ciprofloxacin HCl (20 µg/mL solution) and distilled water were used as the positive and negative controls, respectively. Approximately 40 µL of AgLTF was loaded into different wells using a micropipette for sample testing. The plates were incubated at 37 °C for 18 h. The diameter of the inhibition zones was measured using a plastic ruler. The measurements were made in triplicate.

#### 2.12.4. Disk Diffusion Method

The disk diffusion test was used to evaluate the antimicrobial properties of the prepared hydrogels. Similar concentrations of bacterial suspensions (1 × 10^8^ CFU/mL) were uniformly spread onto the surface of MHA on agar plates. Filter papers (particle retention 11 µm) were cut into small disks with dimensions of 6 mm diameter and 180 µm thickness. Disk-shaped filter papers were then placed aseptically onto the agar. DL and SL hydrogels (70 µL) were carefully transferred on the disks using a sterile spatula and incubated at 37 °C for 18 h. A blank gel containing only gelatin (3% *w*/*v*) and genipin (0.005% *w*/*v*) in distilled water was used as the negative control, and Ciprofloxacin disk (5 µg) was used as the positive control. The assay was conducted in triplicate.

### 2.13. Anti-Biofilm Potential

The anti-biofilm properties of the respective active agents were evaluated using the crystal violet assay by measuring biofilm inhibition [58]. First, a standardized bacterial suspension was acquired by spectrophotometrically adjusting the absorbance at 560 nm to 0.02. One hundred microliters of each culture was transferred to the wells of a flat-bottomed 96-well plate and incubated for 24 h without shaking for biofilm formation. Then, 100 µL of solutions containing AgLTF (Ag/LTF: 1000/2000, 500/1000, 250/500, 125/250, 6.25/125, and 32/62.5 µg/mL) and LTF (2000, 1000, 500, 250, 125, and 62.5 µg/mL) at six different concentrations were added to the wells and further incubated at 37 °C for 24 h. Sterile distilled water was used as the negative control. After the second incubation, the 96-well plates were washed three times with distilled water and dried in an oven at 40 °C for 45 min. Then, 100 µL of 1% crystal violet solution was added to the wells and incubated at RT for 15 min. Then, the plates were re-washed three times with distilled water to remove the excess purple stain of crystal violet. Biofilms were then observed as a thin layer of purple gel-like films attached to the side and bottom of the wells. Subsequently, 150 µL of ethanol was added to destain the wells. Finally, a 100 µL aliquot of the destaining solution from each well was transferred to a new plate. 

As for the hydrogel samples, a similar method was used, except that the crystal violet assay was conducted in a 6-well plate to accommodate the gels. One milliliter of bacterial cultures were added to their assigned wells and incubated until the biofilm formed. After the first incubation, the MHB media containing the bacterial cultures were replaced with 1 mL of fresh MHB containing AgNPs, SL, and DL hydrogels (approximately 1 mL of gel volume). The plates were incubated for another 24 h. The wells were then washed, dried, and stained with a crystal violet solution. The plates were re-washed and destained with 1 mL ethanol. One milliliter of the destaining solution from each well was also transferred to a new plate for further evaluation. The absorbance of all samples was recorded at 590 nm using a microplate reader (Thermo Scientific Multiskan GO, Thermo Fisher Scientific, MA, USA). The mean absorbance for each sample was determined, and the percentage of biofilm inhibition was calculated using Equation (3) below [59]:

(3) $$\mathrm{Biofilm}\;\mathrm{inhibition}\;\%=\frac{\mathrm{Control}\;\mathrm{OD}590\mathrm{nm}-\mathrm{Treatment}\;\mathrm{OD}590\mathrm{nm}}{\mathrm{Control}\;\mathrm{OD}590\mathrm{nm}}\;\cdot \;100$$

After the assay, unstained biofilms (post-treatment) were collected and dispersed in 5 mL of MHB using an ultrasonicator for 15 min at 40 Hz. 0.1 mL of dispersed biofilms were diluted in 9.9 mL of MHB (1:10 ratio) and spread uniformly onto a dried agar surface using a sterile loop. Diluted suspensions were plated on the MHA and incubated for 24 h before calculating the CFU.

### 2.14. In Vitro Cytotoxicity and Efficacy Testing of HaCaT

#### 2.14.1. Cell Viability Determination

The AB cell viability assay reagent was used in this test. HaCaT cells (1.0 × 10^4^ cells/well) were seeded in a 96-well plate containing 100 µL of DMEM and incubated for 24 h until they reached 50% confluency. All cells were maintained in an incubator at 37 °C in a humidified 5% CO2/95% air atmosphere. After the 24 h incubation period, the cells were treated with 20 µL SL containing various concentrations of AgNPs (125, 250, and 500 µg/mL), LTF (250, 500, and 1000 µg/mL), and DsiRNA (15, 30, and 60 ng/mL). A blank media without cells was used as the negative control, whereas untreated cells in DMEM were used as the positive control. Then, 12 µL of AB reagent (10% of the volume in the well) was aseptically added to the wells, and the cells were further incubated for 72 h. The absorbance of each sample at 570 and 600 nm was measured spectrophotometrically using a microplate reader (Biotek PowerWave XS, Marshall Scientific, NH, USA) at 24, 48, and 72 h. 

The number of viable cells was expressed as a percentage of AB reduction. The percentage of AB reduction (AB reduction %) was determined using Equation (4) below [60]:

(4) $$\mathrm{AB}\;\mathrm{reduction}\;\%=\frac{\left(ℇ\mathrm{ox}ℷ2\right)\left(Aℷ1\right)-\left(ℇ\mathrm{ox}ℷ1\right)\left(Aℷ2\right)}{\left(ℇ\mathrm{red}ℷ1\right)\left(A\prime ℷ2\right)-\left(ℇ\mathrm{red}ℷ2\right)\left(A\prime ℷ1\right)}\;\cdot \;100$$

In the formula, ℇℷ1 and ℇℷ2 are constants representing the molar extinction coefficient of AB at 570 and 600 nm, respectively, in the oxidized (ℇ ox) and reduced (ℇ red) forms. The constant values are 117,216 (ℇoxℷ2), 80,586 (ℇoxℷ1), 155,677 (ℇredℷ1), and 14,652 (ℇredℷ2). Aℷ1 and Aℷ2 represent the absorbance of the test wells at 570 and 600 nm, respectively. A′ℷ1 and A′ℷ2 represent the absorbance of the negative control wells at 570 and 600 nm, respectively. The values of AB reduction % were corrected for background values of negative controls containing medium without cells.

#### 2.14.2. Cell Migration Assay

Before testing, HaCaT cells were cultured in low glucose DMEM (10% FBS) in T75 flasks until full confluency was reached. The treatment groups were split between cells exposed to low and high glucose. In the low glucose treatment group, cells were seeded in 6-well plates (4.0 × 10^5^ cells/well) in low glucose DMEM and pre-incubated until 90–95% confluency was reached. As for the high glucose treatment group, cells were seeded (5.0 × 10^5^ cells/well) similarly, except that high glucose DMEM was pre-incubated for 48 h prior to the assay. Subsequently, a monolayer of cells formed was washed with PBS, and a scratch was made using a sterile 200 µL micropipette tip at a predetermined line in each well. PBS was then replaced with fresh low and high glucose DMEM (1% FBS) according to their treatment groups to remove cell debris. Two milliliter hydrogels (blank, AgNPs, AgLTF, SL, and DL) were placed in a cell strainer with a pore size of 70 µm and slowly added into each well containing 2 mL media (1:1 ratio). Growth media (DMEM) without hydrogel was used as a control. Finally, the cells were incubated further for another 72 h. The cell migration was viewed under an inverted phase contrast microscope (Olympus CK30, Tokyo, Japan) connected to a digital camera (Xcam-α) using DigiAcquis version 2.0 software (Matrix Optics, Petaling Jaya, Malaysia). Images of cells within the scratch area were taken at 0, 24, 48, and 72 h. The migration rate (%) is calculated using Equation (5) below [61]:

(5) $$\mathrm{Migration}\;\mathrm{rate}\;\%=\frac{A0\;\left(\mathrm{Initial}\;\mathrm{scratch}\;\mathrm{distance}\right)-\mathrm{At}\;\left(\mathrm{Remaining}\;\mathrm{scratch}\;\mathrm{distance}\;\mathrm{at}\;\mathrm{time}\;t\right)}{A0}\;\cdot \;100$$

#### 2.14.3. Measurements of PGE_2_ Protein in HaCaT Cells

Protein was extracted from the cells after 72 h of treatment by scraping the cells in a cold RIPA buffer-PMSF (500/0.5 µL per well) cocktail. Scraped cells in each well were aspirated into centrifuge tubes and placed in an ice-filled container for 30 min to 1 h to start the cell lysis process. The tubes were intermittently shaken every 5 min using a vortex instrument to ensure that the cell lysate was homogenized during lysis. Then, the tubes were centrifuged at 10,000 rpm and 4 °C for 10 min. The supernatant was pipetted into an Eppendorf tube and stored in a −80 °C freezer until the assay. PGE_2_ levels were measured using an ELISA kit (FineTest, Wuhan, China). Briefly, 50 µL of standard dilution buffer (blank), standard, and sample solutions were added to the assigned wells. Then, 50 µL of biotin-labeled antibody working solution was added to each well and incubated for 45 min at 37 °C. After 45 min of incubation, the plate was washed 3 times with wash buffer. Then, 100 µL of horseradish peroxidase (HRP)-streptavidin conjugate working solution was added to the wells, followed by incubation for 30 min at 37 °C. Once incubated, the plate was re-washed 5 times, and 90 µL of 3,3′,5,5′-tetramethylbenzidine substrate, the substrate for HRP, was added to each well. The plate was then incubated in the dark at 37 °C for 20 min until a notable color change to pale blue was observed in each well. Lastly, 50 µL of stop solution was added to all the wells, and the color turned yellow immediately. The absorbance of each sample at 450 nm was measured spectrophotometrically using a microplate reader. The target concentrations of the samples were interpolated from the standard curve.

### 2.15. Statistical Analysis

The data acquired are presented as the mean ± standard deviation. The data were analyzed by one-way ANOVA followed by Tukey’s post-hoc test. Analyses were computed using jamovi. Values of *p* < 0.05 were considered to indicate a statistically significant difference between the samples tested.

## 3. Results and Discussion

### 3.1. Formation of AgLTF

**Figure 1:**
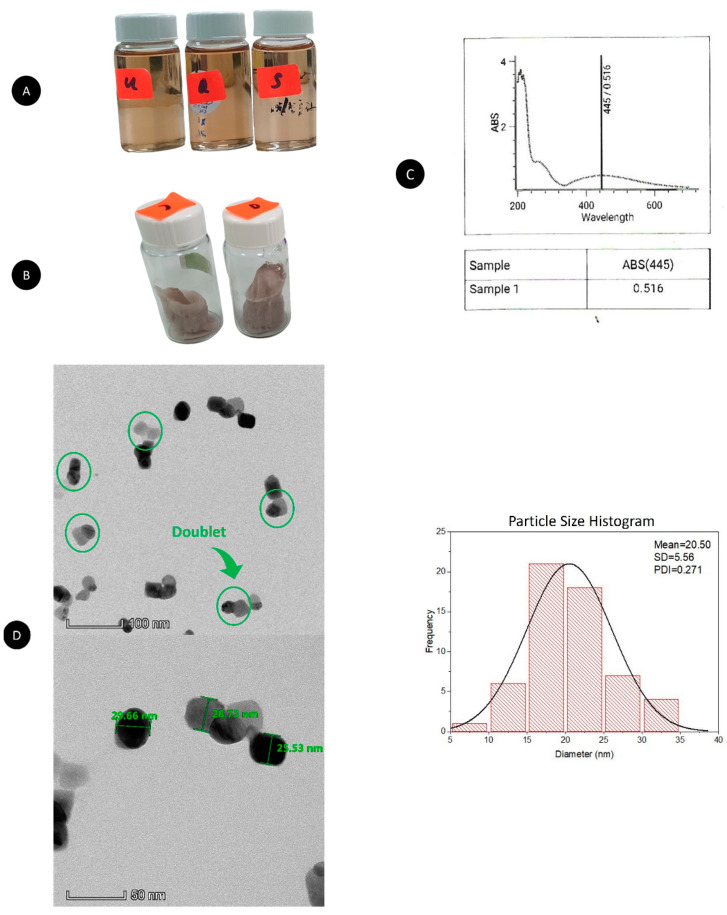
(**A**) Color changes in the reaction mixture of AgLTF, (**B**) Physical appearance of lyophilized AgLTF, (**C**) UV-vis spectrophotometry of AgLTF, and (**D**) TEM micrograph of AgLTF at 260 kx (**top**) and 600 kx (**bottom**) and its corresponding particle size distribution.

AgNPs were formed after 3 days using WETMM as a reducing agent. A gradual color change from colorless to pale yellow and finally to brownish red indicated the formation of an AgNP suspension due to the biomolecules contained in WETMM that facilitated the conversion of Ag^+^ from AgNO_3_ to Ag^0^ (Figure 1A) [62]. After successful conversion into AgNPs, CS played a crucial role in stabilizing the AgNPs from an agglomerated state by forming a monolayer around the AgNPs [53]. Incubation between AgNPs and LTF to form AgLTF did not show any color changes of brownish-red AgNPs, as well as their lyophilized form (Figure 1B); hence, other tests were used to identify the presence and interactions between the two components. UV-vis spectrophotometry was used as a primary analysis to detect the formation of NPs due to the intense surface Plasmon resonance and LTF (Figure 1C) [7]. The typical Plasmon resonance band can confirm the formation of AgNPs at 445 nm, corroborating another publication using an extract of edible mushrooms as a reducing agent [63]. A small peak at the 270–300 nm area indicates the presence of LTF in the AgLTF complex [54]. 

When viewed under TEM, AgLTF complexes were seen to be well dispersed (PDI 0.271) particles and have an average size of 20.50 nm ± 5.56 (Figure 1D). The high presence of primary amines in CS (75–85% deacetylation degree) contributes to the overall positive charge of CS monolayers around AgNPs, which causes a stabilization effect by repelling against each other [64]. Consequently, this minimizes AgNP particles from agglomerating into larger sizes. In addition, positive charges from the primary amines of CS also allow for anionic molecules to form complexes [65]. AgNPs and LTF were presumably complexed via electrostatic interaction between the anionic thiol groups of LTF and cationic amine groups of CS. AgLTF appeared in doublet form (green circle), which could indicate the successful complexation of AgNPs and LTF. Some doublets were also seen grouped, probably due to the diminished stabilizing effect of CS since LTF occupied the primary amines on the chemical structure of CS. AgLTFs were generally spherical with some presence of cubes. Spherical AgNPs obtained through controlled thermodynamic conditions could offer advantages such as stability due to the minimum availability of surfaces [66].

### 3.2. FTIR Analysis

Interactions between AgNPs, WETMM (reducing agent), and CS were established using FTIR [53]. WETMM possessed free aldehyde groups that served as electron donors to reduce metal ions into Ag^0^, whereas CS molecules formed C-O-C bridges as a protective layer around AgNPs to prevent aggregation of NPs [67]. 

**Figure 2:**
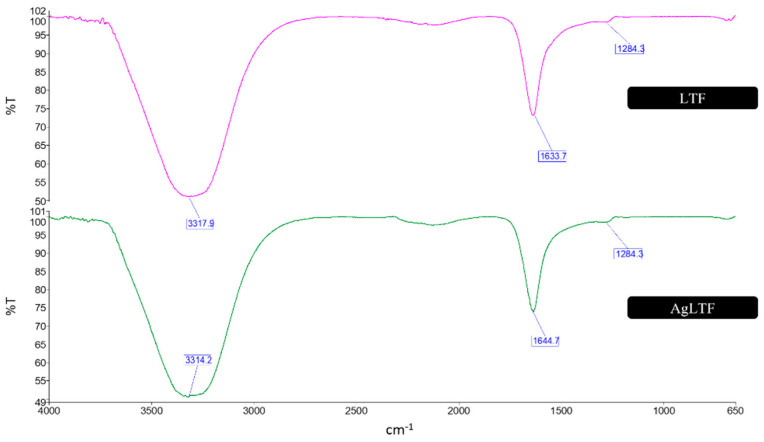
FTIR spectrum of LTF and AgLTF.

In this study, LTF was complexed with AgNPs, and the interactions between the functional groups of these components were examined (Figure 2). LTF was individually presented with a broad band at 3317.9 cm^−1^, representing aromatic C-H compounds and O-H stretching. LTF has large amine groups in its chemical structure, which usually appear around 3300 cm^−1^. Primary amine (RNH_2_) groups typically form a band with two sharp spikes on IR spectra; however, it is overshadowed by the dominant (four times more in abundance) secondary amine (R_2_NH), forming a broad band around the 3300 cm^−1^ area. The sharp band at 1633.7 cm^−1^ represents C=O stretching from the amide and ring C=C vibrations from benzene. The small band at 1284.3 cm^−1^ could also be due to the primary and secondary amide groups.

As for the spectrum of AgLTF, there were mainly overlapping absorption bands from AgNPs and LTF. The strong band at 3314.2 cm^−1^ represents the aromatic C-H compounds from the aromatic hydrocarbons and O-H stretching from the primary amines, primary hydroxyl, and secondary hydroxyl groups. The band at 1644.7 cm^−1^ corresponds to C=C stretching from the ring structure, whereas the subdued 1284.3 cm^−1^ band corresponds to C-O-C bending within the ring structure, and the C-O-C bridges formed during the stabilization process. There were no changes in the functional groups in AgLTF after LTF was incorporated into the AgNPs. This suggests that the complexation between AgNPs and LTF was held together by weak non-covalent electrostatic interactions.

### 3.3. X-ray Diffraction Analysis

**Figure 3:**
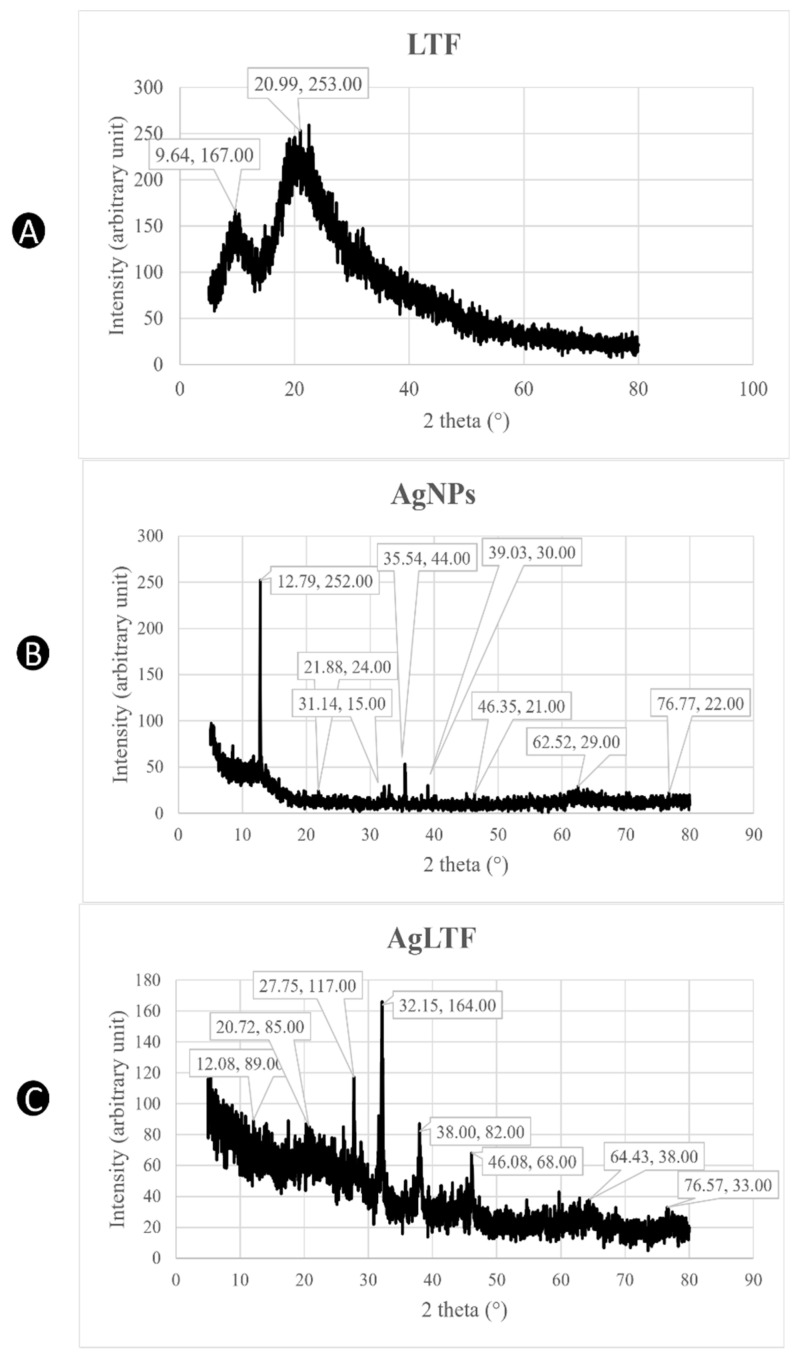
XRD spectrum of lyophilized (**A**) LTF, (**B**) AgNPs, and (**C**) AgLTF.

XRD analyses were incorporated in this study to confirm the crystalline nature of AgLTF [7]. Figure 3A shows two broad humps without sharp diffraction peaks at 2 theta 9.64° and 20.99°, indicating characteristic XRD patterns for pure protein in amorphous forms [68,69,70]. On the other hand, CS from the AgNPs spectrum has a linear semi-crystalline structure with sharp peaks at 2 theta 12.79° from the (020) planes and 21.88° from the (110) planes of ordered crystalline units (Figure 3B) [71]. Tall peaks at 2 theta 31.14° and 35.54° represent the crystallization of the bio-organic phase occurring on the surface of the AgNPs [72]. The presence of subdued peaks at 39.09°, 46.35°, 62.52°, and 76.77° indicates that AgNPs are face-centered cubic and crystalline in nature [73,74], consistent with those found in the Joint Committee on Powder Diffraction Standards database. The relative intensity of these peaks is due to the ratio of CS and AgNPs being used to formulate the stabilized AgNPs, which was supported by the wide scan XPS spectrum, which revealed the atomic percentages of O, C, N, and Ag to be 34.64%, 57.88%, 4.92%, and 2.56%, respectively [53]. In Figure 3C, the two peaks at 2 theta 9.64° and 20.99° that were present in the LTF spectrum became very weak once they complexed with AgNPs due to the crystal formation of the product via freeze-drying during the drying process [75]. The characteristic peaks of CS were also significantly decreased as a result of the complexation process with LTF due to its excellent sorption ability [76]. The highly deacetylated structure and low molecular weight of CS allow for the adsorption of hydrophilic moieties of LTF onto the hydroxyl and amine groups of CS [77]. The characteristic peaks for AgNPs were shifted, probably because of the change in AgNPs (stabilized by CS) structure geometry due to interaction or complexation with LTF [78]. More importantly, the peaks at 2 theta 38.00°, 46.08°, 64.43°, and 76.57° together with their relative intensity can be assigned to the planes of (111), (200), (220), and (311), which show the crystalline structure of AgLTF [73,74].

### 3.4. DsiRNA Adsorption to AgLTF

The binding of DsiRNA to AgLTF was achieved via simple complexation. As shown in Table 1, AgNPs have a high zeta potential of +31.7 ± 4.8 mV, primarily due to the protonated amino groups in CS. LTF has a molecular size of 80 kDa, whereas DsiRNA has a molecular size of 16 kDa. When AgNPs were complexed with LTF and DsiRNA, the particle sizes progressively increased from 58.4 ± 6.3 nm to 113.3 ± 25.0 nm and finally to 157.4 ± 5.0 nm, which could indicate successful complexation between the compounds. The zeta potential was also decreased from +31.7 ± 4.8 mV to +18.3 ± 1.5 mV and further reduced to +16.3 ± 12.3 mV, probably due to the occupation of cationic amine groups of CS with anionic groups of LTF and later with DsiRNA. The low PDI of AgLTF and AgLTF-DsiRNA showed that they were homogenous and moderately dispersed [79].

EE was measured to determine the DsiRNA carrying efficiency of AgNPs and AgLTF [80]. According to Table 2, two concentrations (500 and 125 µg/mL) of AgLTF and AgNPs were able to load with DsiRNA at moderate EE levels [72% and 70% for 500 and 125 µg/mL, respectively, and they were not statistically significant (*p* > 0.05, F = 0.934)], which corroborates the previous finding that different concentrations of gold nanoparticles (AuNPs) did not affect the entrapment capacity of DsiRNA [35]. In fact, increasing the concentration of the carrier molecule might have a negative effect on EE [39,80]. AgLTF and AgNPs-DsiRNA only achieved 70–72% EE, probably due to the shielding effects and steric hindrance, which interfered with the interaction between negatively charged DsiRNA molecules and positively charged amine groups of CS [39].

**Figure 4:**
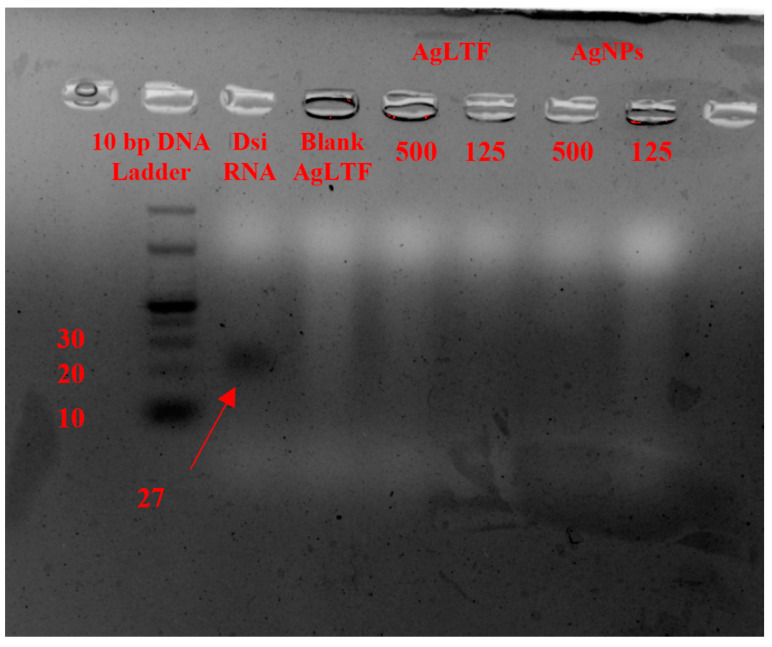
Gel electrophoresis of AgLTF-DsiRNA and AgNP-DsiRNA at 500 and 125 µg/mL.

The gel electrophoresis method with 5% agarose and red gel staining was used to analyze the DsiRNA binding efficiency to AgLTF. The black trailing bands in Figure 4 represent the DNA being electrophoresed along the agarose gel. Generally, the heavier the molecule, the shorter the distance it travels. On this basis, the column DNA Ladder highlights the position of DNA with different molecular weights based on its number of base pairs. With this as a reference, the naked DsiRNA against the PGT gene (27 base pairs) acted as the positive control and traveled to the position between 20 to 30 bp DNA, whereas blank gel (without DsiRNA) served as the negative control. Hydrogel samples loaded with various concentrations of AgLTF and AgNPs (500 and 125 µg/mL) and 15 ng/mL DsiRNA showed no trailing bands of DsiRNA after being electrophoresed, which demonstrated that there was strong binding between DsiRNA and AgLTF and AgNPs. These findings indicate that AgNPs could be complexed and carry sufficient DsiRNA to the wound site. Moreover, DsiRNA carried by AgNPs (stabilized by CS) was reportedly more likely to be taken up by cells through the endocytosis process due to the electrostatic interaction of CS with cellular membranes [81].

### 3.5. Formation of the Hydrogel

**Figure 5:**
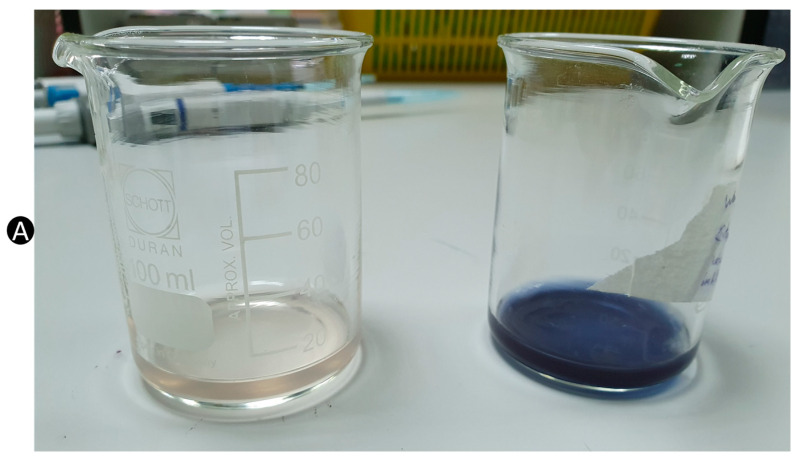

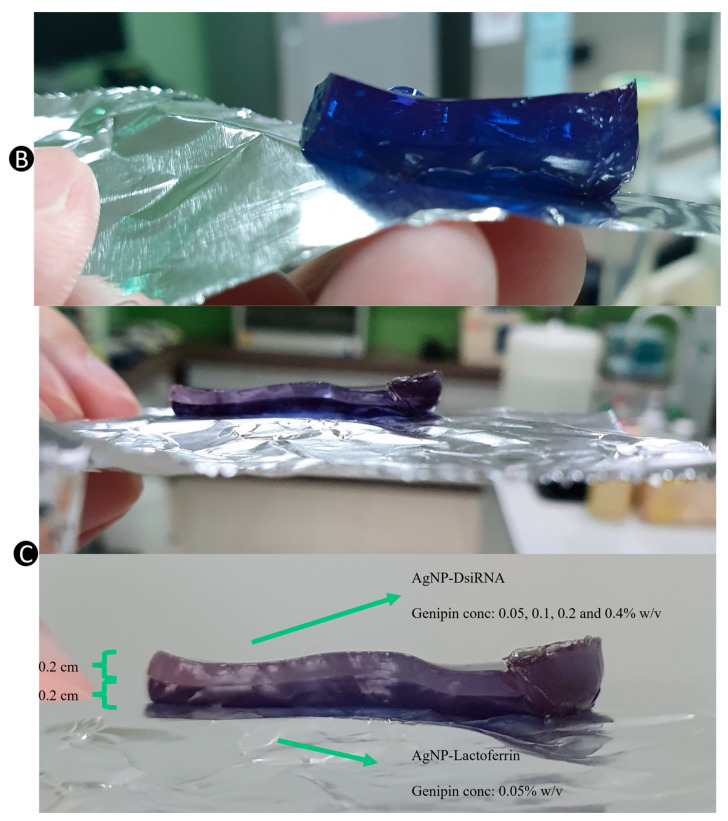
(**A**) Formation of hydrogel indicated by the color change, (**B**) Cross-sectional view of the formed SL hydrogel, and (**C**) Cross-sectional view of the formed DL hydrogel.

After 24 h incubation, the pale brownish color of the nanocomposites and gel polymer mixture turned dark blue, indicating successful gelation (Figure 5A). A blue-colored SL hydrogel was obtained when removed from the beaker (Figure 5B). As for DL hydrogel, when it was extracted, a clear separation between the upper and bottom layers containing two different complexes of active components was observed (Figure 5B). The extracted hydrogel had a consistent dimension of 0.4 cm height (0.2 cm height for each layer), with each layer holding a 6 mL volume. Gel was constructed in such a way because sequentially treating infected wounds in each critical stage of the wound healing process resulted in a better healing effect [51]. Usually, when a skin layer is damaged, inflammation starts immediately and can last for a week, whereas proliferation only occurs from day 2 up to day 10 [51]. Hence, AgNPs-DsiRNA was compartmentalized in the upper layer to achieve delayed and slow release from the hydrogel to synchronize with the biological angiogenesis process, preceded by immediate release of AgLTF in the bottom layer for fast eradication of biofilm-producing pathogens during the inflammation stage.

### 3.6. Characterization of the Hydrogel

According to Table 3, all of the hydrogels formed using various concentrations of genipin (0.005, 0.01, 0.02, and 0.04% *w*/*v*) displayed mostly similar physical characteristics, except for the lowest genipin concentration (0.005% *w*/*v*). Hydrogels consisting of genipin 0.005% *w*/*v* in the upper layer were bouncy on touch, in contrast to the other hydrogels with higher concentrations of genipin, forming rigid and very sticky hydrogels. This supports the previous finding that the viscoelasticity of hydrogel can be adjusted by varying the crosslinker concentration [54]. As the genipin concentration increases, the tensile strength of the hydrogel increases. Sufficient hardness is often needed to withstand handling during packaging and administration without disrupting the integrity of the gel. However, excessive hardness and stickiness may cause undesirable effects, as removing the gels from the glass beakers and cutting them into smaller pieces would be more challenging. In addition to having a rigid structure, many samples were wasted while handling hydrogels with genipin concentrations higher than 0.005% *w*/*v* (0.01, 0.02, and 0.04% *w*/*v*). Thus, genipin 0.005% *w*/*v* in the upper layer of the DL gel was selected for further tests.

**Figure 6:**
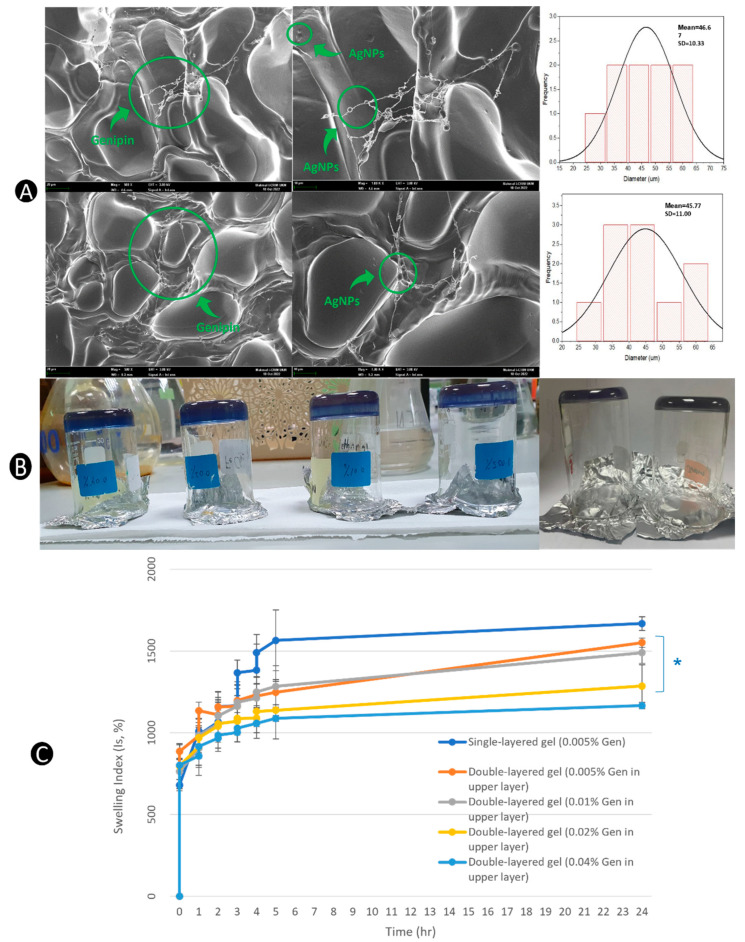
(**A**) SEM micrographs of the upper layer (**top**) and lower layer (**bottom**) of hydrogel with genipin (0.005% *w*/*v*) containing AgLTF at 0.5 kx (**left**) and 1.0 kx (**right**), (**B**) Adhesion test of DL hydrogel samples containing various concentrations of genipin (0.005%, 0.01%, 0.02%, and 0.04%) and SL hydrogel (genipin 0.005%), and (**C**) Swelling index over time graph of hydrogel samples, *n* = 3. * statistically non-significant difference from the single-layered gel (0.005% genipin) (*p* > 0.05).

SEM micrographs showed interlinking chains between gelatin polymers, which created 3D microporous structures in the hydrogels. Gelatin is a polypeptide with high molecular weight and many functional groups, so it can readily be crosslinked with [40]. A previous report showed that uncrosslinked gelatin (3% *w*/*v*) has an average pore size of 105 ± 22 µm [54]. In Figure 6A, gelatin (3% *w*/*v*) crosslinked with genipin (0.005% *w*/*v*) hydrogel appeared to have a 46.67 ± 10.33 µm pore size for the first layer. On the other hand, the second layer of hydrogel had an average pore size of 45.77 ± 11.00 µm, which was similar to the first layer, as they both had the same compositions of hydrogels. Smaller and more consistent pore sizes in crosslinked gelatin hydrogels are a result of the crosslinking effects of genipin. Genipin stabilizes and tightens the hydrogel matrix, reducing the structure’s pore size and flexibility [63]. In SEM, genipin was seen to form interconnective chains between the gel polymers, increasing the mechanical properties and reducing the likelihood of collapsing in media (Figure 6A). Genipin is necessary to maintain the integral stability of gelatin, as uncrosslinked gelatin gels are not stable under physiological conditions (37 °C and pH 7.4) [48]. AgNPs, represented by the spheres in the image, can be seen dispersed within the gelatin polymers and outside the polymers along the interconnectivity chains of genipin. AgNPs can form nanoparticle–hydrogel hybrid systems using strong hydrophobic interactions [50,82]. Through this dynamic, AgNPs can further enhance the mechanical stability of the hydrogel in the matrix system.

The swelling index over time graph demonstrated reduced swelling capacity in a 24-h window period when the genipin concentration in the second layer of hydrogel was increased (Figure 6C). SL hydrogel with 0.005% *w*/*v* genipin achieved the highest swelling index (1668%), whereas DL hydrogel with 0.04% *w*/*v* genipin achieved the lowest swelling index (1166%) due to the smaller pore sizes in hydrogels with higher concentrations of genipin, which restrained the capacity of gelatin peptide chains to undergo relaxation when water was absorbed. When swelling occurs, the hydrogel matrix expands. The expansion in hydrogel causes pore sizes to increase, facilitating the release of AgNPs from the hydrogel [63]. Thus, when the genipin concentration is increased, the hydrogel’s swelling capacity is reduced, which could potentially slow down the release of entrapped drugs. Hence, it is vital to strike a balance when finding a suitable concentration of crosslinker that provides gel stability and timely release of AgNPs.

In this experiment, all prepared formulations exhibited excellent adhesive properties (Figure 6B). Adhesion is an important quality of hydrogels, especially in topical applications, so it remains intact with the skin throughout the treatment process.

### 3.7. In Vitro Drug Release of Active Agents

Gelatin hydrogel follows the principle of swelling-controlled drug release [82]. When in contact with bio-fluid, the hydrogel expands beyond its limit and increases its pore size, facilitating the drug diffusion process along with polymer chain relaxation. In this case, AgNPs, LTF, and DsiRNA diffuse down the concentration gradient during swelling within the hydrogel matrix toward the injury site to exert their effects. The in vitro release profile of each active component was conducted using the Franz cell diffusion system. Many factors affect drug release from a hydrogel, such as pH, temperature, and ionic strength [82]. However, only the pH values of the media were varied (7.4 and 8.0) during the test to mimic the possible pH elevation of human skin tissue during infection [83]. Cellulose acetate membrane of 0.45 µm pore size was used for Franz cell diffusion, as it had been shown to be able to support a wide range of hydrophobic (LTF) and hydrophilic (AgNPs and DsiRNA) samples and applications [84].

**Figure 7:**
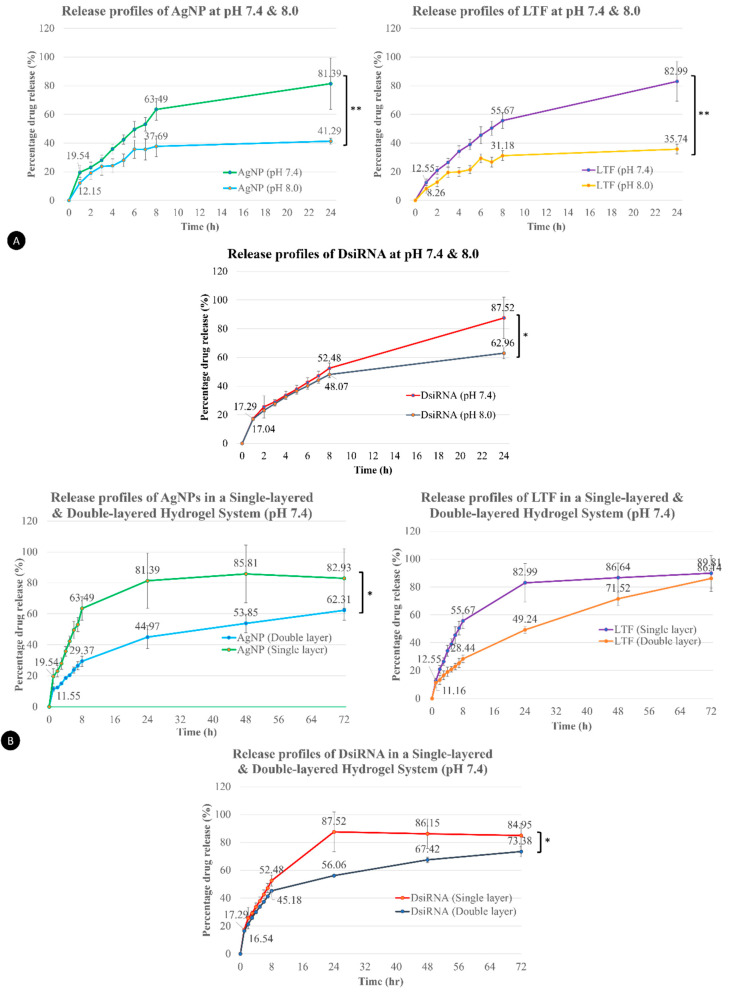
In vitro drug release of AgNPs, AgLTF, and DsiRNA (**A**) at pH 7.4 and 8.0 (**B**) from the SL and DL gelatin hydrogels, *n* = 6. * statistically significant difference (*p* < 0.05); ** statistically significant difference (*p* < 0.01).

In general, drug release behavior from gelatin hydrogels occurs in the following phases: a typical initial burst release followed by diffusion, combined diffusion and degradation, and finally, polymer degradation [40]. In SL hydrogel, burst release was displayed by the steep increase in drug release at the first hour to demonstrate the rapid release of AgNPs (19.54%), LTF (12.55%), and DsiRNA (17.30%) from the hydrogel polymer matrix (Figure 7A). This was followed by a steady release of the actives until finally achieving 81.39% for AgNPs, 82.99% for LTF, and 87.52% for DsiRNA at the 24 h time point. In a previous study, complete DsiRNA release from hydrogel using PF127 occurred within an 8 h window period [35]. At pH 8.0, all active ingredients in the hydrogel demonstrated slower and incomplete release of 52.77% for AgNPs, 35.74% for LTF, and 62.96% for DsiRNA. Since gelatin polymers crosslinked with genipin were maintained in the gel form throughout the process, its charge groups were probably not disrupted to affect the drug release of entrapped components. However, the cationic nature of CS, which is responsible for enveloping AgNPs and forming complexes with LTF and DsiRNA, could be affected. In a higher pH medium, the basic amine groups of CS were not protonated. Thus, there were fewer positively charged ions to repel and cause swelling to facilitate drug release [85].

As for the DL hydrogel, the Franz cell diffusion study was extended up to 72 h (Figure 7B). During the first hour, AgNPs achieved 11.55% drug release and 11.16% and 16.54% LTF and DsiRNA, respectively. The initial release of these components was reduced in the DL hydrogel compared to the SL hydrogel. However, they were not significantly different (*p* > 0.05, F = 0.083) as some AgNP-DsiRNA in the upper layer might have diffused into the bottom via pores during storage. At 24 h, only 44.98% of AgNPs, 49.24% of LTF, and 56.06% of DsiRNA were released from the DL hydrogel and finally achieved 62.31% (AgNPs), 71.52% (LTF), and 73.38% (DsiRNA) at 72 h (*p* < 0.05). The drug release profiles of different components (AgNPs and DsiRNA) in DL hydrogel were significantly different from the SL counterpart, except for the release profile of LTF in these formulations, due to the hydrophobic nature of LTF diffusing across a hydrophilic cellulose acetate membrane in the Franz cell diffusion system [86]. Hydrophobic compounds typically have a concave-up drug diffusion pattern across various synthetic membranes, with a slow diffusion at the starting point, which accelerates over time [87]. Compared to DsiRNA, at 72 h, LTF release was a total of 86.14%, which is higher than 73.38% for DsiRNA despite the slow start within 24 h (49.24% for LTF vs. 56.06% for DsiRNA) of the diffusion process. Hence, there was no significant difference in the release profiles for LTF in DL and SL hydrogel because of the compound’s physicochemical properties and the membrane used [87]. Although only DsiRNA was intended to have a prolonged release in the DL hydrogel, AgNPs and LTF were also impacted, probably due to the equilibrium process in the first and second layers of the DL hydrogel.

### 3.8. Antibacterial Activity

**Figure 8:**
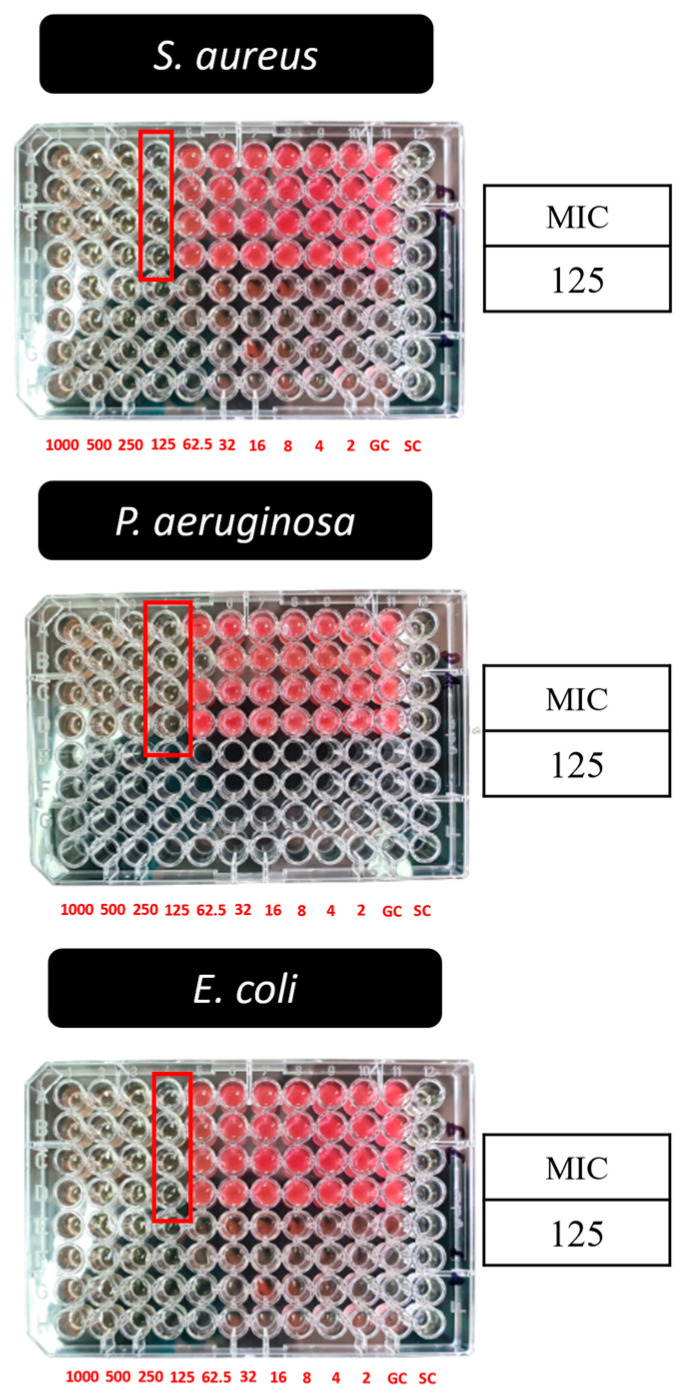
MIC of AgLTF against *S. aureus*, *P. aeruginosa,* and *E. coli,* as determined by the microbroth dilution assay, *n* = 4.

In this study, microbroth dilution and agar well diffusion methods were employed to determine the antibacterial effects of AgLTF in solution, while the disk diffusion assay was for the gel form. SL and DL gels were tested against the three most common bacterial pathogens found in diabetic wounds or foot infections: *S. aureus*, *P. aeruginosa,* and *E. coli* [88]. In the microbroth dilution method using gels containing AgLTF, starting from concentrations 1000 to 2 µg/mL, all three tested organisms had red formazan stains in the wells containing 62.5 µg/mL AgLTF and lower (Figure 8). This indicated that the MIC value of AgLTF was 125 µg/mL, which is consistent with the biosynthesized AgLTF reported elsewhere [54]. The antibacterial efficacy of AgNPs is well established, and the mechanisms by which AgNPs exert their effects are diverse. In the nanoparticle state, AgNPs can accumulate in the pits or edges on the bacteria’s cell wall after they anchor to the cell surface and cause cell membrane denaturation [89], preceded by penetration into bacterial cells due to their small size and damaged organelles [11]. In addition, AgNPs can also release Ag^+^_,_ the killer for microbes. Ag^+^ has an electrostatic affinity toward the cell wall and cytoplasmic membrane, disrupting the bacterial envelope. When Ag^+^ enters the cell, respiratory enzymes can be deactivated due to their electrostatic interaction with thiol groups and cause the generation of reactive oxygen species. Additionally, Ag^+^ can also prevent the synthesis of proteins by denaturing ribosomes in the cytoplasm [11]. The efficacy of AgNPs as antibacterial agents also depends on their morphology. AgNP crystals are more likely to attach to the surface of the cell membrane due to their reactive (111) facets [90]. Spherical and smaller AgNPs are also prone to release more Ag^+^ for their toxic effects against bacterial pathogens [11].

Agar well diffusion was conducted to investigate the antibacterial properties of AgLTF and LTF alone. Zones of inhibition from this assay are presented in Table 4. AgLTF at a concentration of 125 µg/mL formed the biggest zone of inhibition against *E. coli* (11.3 ± 7.5 mm), followed by *S. aureus* (10.3 ± 1.5 mm) and *P. aeruginosa* (7.3 ± 0.6 mm) (*p* < 0.05). AgNPs have more activity against Gram-negative bacteria due to the thin layer of peptidoglycan (PG) cell wall and the presence of lipopolysaccharides (LPS), which makes it easier for positively charged CS and Ag^+^ to bind to [27]. However, inhibition zones against *P. aeruginosa* were lower than both *E. coli* and *S. aureus* because they are more resistant to AgNPs [90]. When the concentration of AgLTF was increased to 1000 µg/mL, the zones of inhibition were also increased to 13.3 ± 2.9 mm for *E. coli*, 11.3 ± 1.2 mm for *S. aureus,* and 9.3 ± 0.6 mm for *P aeruginosa*, which indicates that the antibacterial effect is dose-dependent. Although recombinant human LTF derived from rice and human milk LTF were reported to have a broad spectrum of antibacterial effects [91,92], the LTF (1000, 500, 250, and 125 µg/mL) used in this study demonstrated nil antibacterial effects against *E. coli*, *S. aureus,* and *P. aeruginosa*. This finding is consistent with a previous report that LTF exerted no antibacterial effects against *E. coli*, *S. aureus*, *Bacillus* sp., and *P. aeruginosa* [27].

A disk diffusion assay was conducted to determine the antibacterial properties of AgLTF after it was formulated into a hydrogel. This method is preferred when assessing the antibacterial effects of hydrogels because it allows active components to diffuse from the gel matrix through a filter paper disk and disperse into agar, which better mimics a topical application onto the skin. In contrast, agar well diffusion was more suited to extract solutions [93]. SL gel demonstrated the highest activity against *E. coli* (13.3 ± 0.6 mm) and *P. aeruginosa* (13.3 ± 0.6 mm), followed by *S. aureus* (10.3 ± 1.5 mm). Similarly, DL gel also showed the highest activity against *E. coli* (15.0 ± 1.0 mm), followed by *P. aeruginosa* (14.7 ± 0.6 mm) and *S. aureus* (10.0 ± 1.7 mm). The difference between the antibacterial activity of SL and DL was insignificant (*p* > 0.05, F = 1.93). The results indicate that the main antibacterial component (AgNPs) was not impacted by the gel matrix and could diffuse through the agar and exert antibacterial effects.

### 3.9. Anti-Biofilm Potential

This study employed a microtiter plate assay using crystal violet stain to quantitatively determine the anti-biofilm potential of SL and DL hydrogels containing AgLTF-DsiRNA. *P. aeruginosa* and *S. aureus* were selected as the test organisms, as they are known biofilm formers [22]. Previously, AgNPs were tested against these two microbes, and they showed inconsistent anti-biofilm activity [53]. Some of the higher doses of AgNPs were shown to have decreased anti-biofilm activity, partly due to the sub-bacteriostatic concentration of LMW CS (0.09% *w*/*v*), which might have triggered the stress response mechanism of the bacteria [94]. Furthermore, another study reported the pro-biofilm activity of certain antibiotics and NaCl when high doses were used due to the increased expression of polysaccharide intercellular adhesin, a component of the biofilm matrix [95]. Hence, this negative anti-biofilm effect might be a coping mechanism for bacteria to increase their survivability against these toxic agents. Much of the anti-biofilm effects were due to the nanoscale feature of AgNPs, which can easily penetrate the complicated architecture of biofilm [11]. However, findings from other reports have concluded that the anti-biofilm properties of AgNPs are ultimately inconclusive and strain-dependent [11,28,54].

**Figure 9:**
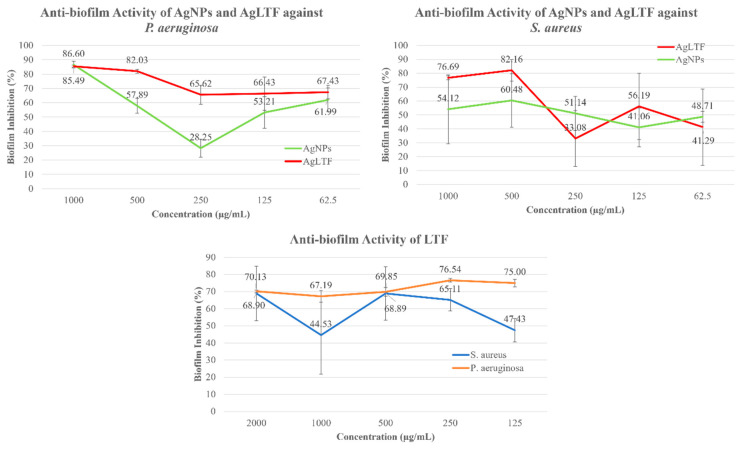
Anti-biofilm activity of AgNPs, LTF, and AgLTF against *S. aureus* and *P. aeruginosa* using the crystal violet assay, *n* = 3.

In this study, LTF was incorporated with AgNPs to enhance the overall anti-biofilm effects of this complex. When tested against *S. aureus*, LTF in solution form demonstrated an increase in anti-biofilm activity from 47.43 ± 6.95% at 62.5 µg/mL to 68.90 ± 16.01% at 1000 µg/mL, with an exception for concentration 500 µg/mL where it displayed a dip (44.53 ± 22.62%) in anti-biofilm activity (Figure 9). A significant jump in biofilm inhibition % was observed when the concentration of LTF was increased from 125 µg/mL (47.43 ± 6.95%) to 250 µg/mL (65.11 ± 6.50%) (*p* < 0.05). The increase in activity from 250 µg/mL to 500 µg/mL (68.89 ± 15.62%) and 2000 µg/mL (68.90 ± 16.01%) was not significant (*p* > 0.05, F = 1.55). Interestingly, LTF at 125 and 250 µg/mL concentrations had higher anti-biofilm activity than the positive control (*p* < 0.05), highlighting the importance of using lower doses for AgNPs and LTF to avoid the negative or stress feedback mechanism of the bacteria. As for *P. aeruginosa*, the anti-biofilm activity of LTF was remarkably higher than the positive control at all concentrations (2000, 1000, 500, 250, and 125 µg/mL) (*p* < 0.05). LTF had the highest activity (76.54 ± 1.05%) at 250 µg/mL and the lowest activity (67.19 ± 3.39%) at 500 µg/mL. In a previous study, LTF was tested for its anti-biofilm effect against 25 different strains of Group B *Streptococcus* (GBS) [96]. LTF at 250 µg/mL decreased biofilm formation in 20 out of 25 strains of GBS and significantly inhibited biofilm formation in both high and low biofilm formers. On the other hand, LTF at 500 µg/mL decreased biofilm formation of 21 out of 25 strains of GBS, but only inhibited biofilm formation of the high biofilm formers. Similarly, LTF against *S. aureus* and *P. aeruginosa* in this study did not show a dose-dependent relationship with activity and had the most optimal effect at 250 µg/mL. The discrepancy between the activity against Gram-negative and Gram-positive bacteria is probably due to the different matrix types produced by the bacteria. *P. aeruginosa* is known to have a thick matrix composed mainly of polyanionic EPS. In contrast, *S. aureus* has EPS that is polycationic [97]. LTF and AgNPs (stabilized by CS) are highly cationic due to the abundant amine groups present in their structure, which could facilitate the attachment and penetration of these compounds into the EPS matrix of *P. aeruginosa* [32]. LTF is an iron-chelating protein that disrupts the iron regulation of the biochemical and metabolic functions of bacteria, which are essential for growth and development [98]. LTF can prevent lectin-dependent bacterial adhesion or motility to the surface via EPS synthesized by bacteria, which is the first step in biofilm formation [99]. In addition, LTF can exert direct effects by depriving the biofilm of iron nutrients and nullifying the scavenging system of biofilm to gather essential minerals and nutrients, as it can diffuse through the bacterial biofilm [32]. 

When AgNPs and LTF were combined to form AgLTF, the anti-biofilm activity was mostly enhanced compared to AgNPs alone. In a previous study, AgLTF showed the strongest anti-biofilm activity due to their synergism [27]. LTF has also been used with other agents, such as Xylitol, to further improve efficacy against biofilm-producing organisms [32]. Since AgNPs had an MIC value of 125 µg/mL against all three of the tested microbes in the antibacterial assay and LTF produced optimal anti-biofilm effects at 250 µg/mL, AgLTF complex containing 125 µg/mL of AgNPs and 250 µg/mL of LTF was selected for further testing.

**Figure 10:**
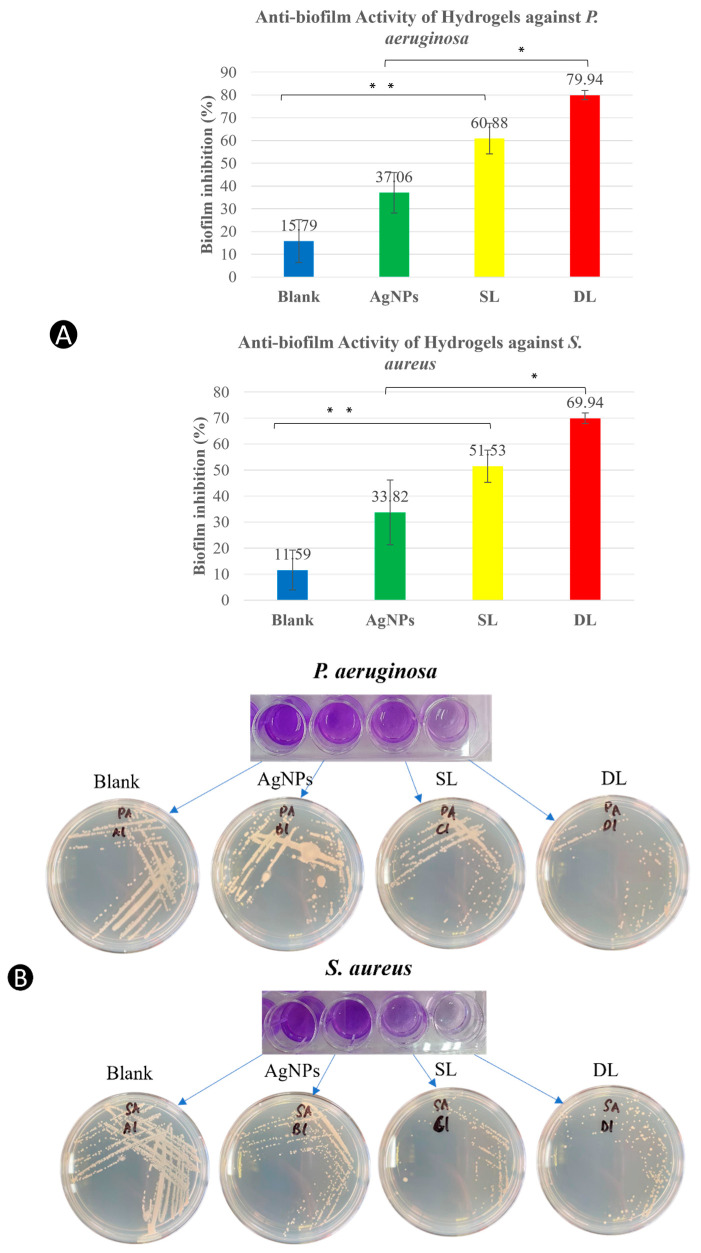
(**A**)Anti-biofilm test of blank, AgNPs, SL, and DL gels using the crystal violet assay, *n* = 4, and (**B**) Image of live bacteria in formed biofilms on MHA plates after treatment with the blank (A1), AgNPs (B1), SL (C1), and DL (D1) gels, *n* = 3. * statistically significant difference from the blank gel (*p* < 0.05); ** statistically significant difference from the DL gel (*p* < 0.05).

In the gel form, all three of the treatment groups (hydrogel containing AgNPs, SL, and DL) had higher biofilm inhibition against both *P. aeruginosa* and *S. aureus* than the blank gel (*p* < 0.05) (Figure 10). SL (51.53 ± 6.14% against *S. aureus* and 60.88 ± 6.74% against *P. aeruginosa*) and DL (69.94 ± 2.00% against *S. aureus* and 79.94 ± 2.03% against *P. aeruginosa*) produced higher anti-biofilm inhibition than gel containing AgNPs alone (33.82 ± 12.43% against *S. aureus* and 37.06 ± 8.85% against *P. aeruginosa*), showing the enhanced anti-biofilm effect of LTF and AgNPs. Interestingly, DL had a higher anti-biofilm effect than SL, prompting the question of the AgLTF release pattern’s role in biofilm inhibition. In contrast to the results obtained, a previous study that utilized usnic acid as an antibacterial and anti-biofilm agent in biodegradable polymers against *S. aureus* demonstrated better dual effects in polymers with a faster release profile [100]. When controlled release of plectasin NZ2114 loaded in a catheter matrix was tested against *S. aureus* biofilm, the highest antibacterial and anti-biofilm effects (highest CFU reduction) at day 1 were observed, with a significant drop in activity at day 7 [101]. Since the anti-biofilm assay was only run for 24 h (LTF release percentage was approximately 49% in DL and 86% in SL) and LTF displayed better activity at lower doses, this could be the reason for the higher biofilm inhibition in DL as compared to SL at the 24 h time point. Further investigation and a longer assay duration are warranted to confirm this anti-biofilm activity over time in AgLTF.

### 3.10. Cell Viability

HaCaT cells, a keratinocyte cell line, were selected for in vitro cell viability and migration testing because keratinocytes and fibroblasts are the main components of granulation tissue. They are also the dominant cells involved in the wound closure mechanism [102]. The AB cell viability assay reagent was used to quantify cellular metabolic activity and thus determine the viable HaCaT cells when cultured with hydrogels containing three different concentrations of AgLTF-DsiRNA. AB cell viability reagent can also determine the proliferation rate of cell lines by measuring it at two or more time points in the sample. This test was chosen explicitly over the classical 3-(4,5-dimethylthiazol-2-yl)-2,5-diphenyltetrazolium bromide (MTT) assay, as it is highly stable and non-toxic to the cells, which offers an advantage for continuous monitoring of cultures over time [60]. Mechanism-wise, AB utilizes an oxidation–reduction indicator that fluoresces and changes color due to the chemical reduction process during cell growth. The oxidized form of water-soluble AB dye is blue in color and non-fluorescent (non-viable), whereas the reduced form of AB dye is fluorescent red (viable). In this study, absorbance was measured at 24, 48, and 72 h to observe the viability and proliferation rate of HaCaT cells under the influence of the hydrogels tested.

**Figure 11:**
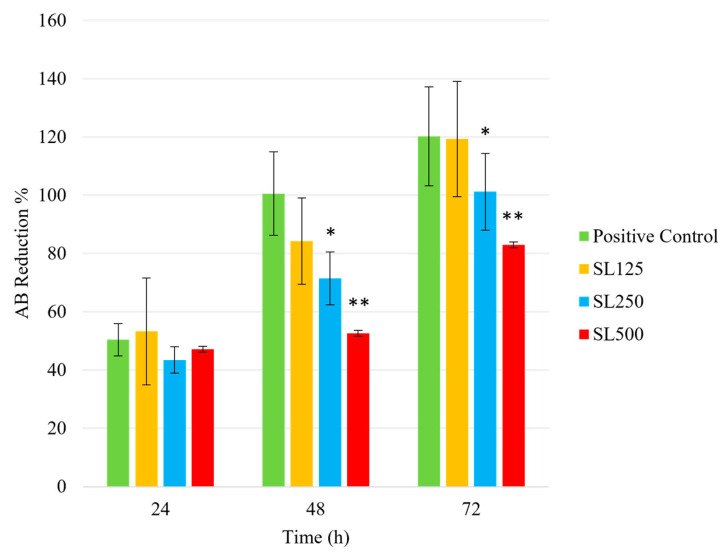
Cytotoxicity effects of SL at different concentrations using the AB assay, *n* = 5. * statistically significant difference from positive control (*p* < 0.05); ** statistically significant difference from positive control (*p* < 0.01).

Figure 11 demonstrates the cytotoxic effects of AgLTF-DsiRNA hydrogels on HaCaT cells. SL125 denotes AgLTF-DsiRNA in SL gel containing 125 µg/mL AgNPs, 250 µg/mL LTF, and 0.015 µg/mL DsiRNA, whereas SL250 represents AgLTF-DsiRNA containing 250 µg/mL AgNPs, 500 µg/mL LTF, and 0.015 µg/mL DsiRNA and lastly SL500 refers to AgLTF-DsiRNA containing 500 µg/mL AgNPs, 1000 µg/mL LTF, and 0.015 µg/mL DsiRNA. At 24 h, all gels were comparable to the positive control in the range of AB reduction of 47.24–53.33% (*p* > 0.05). However, SL250 and SL500 were significantly lower in AB reduction compared to the positive control (*p* < 0.05) at 48 h and 72 h. Hence, SL125 remained non-cytotoxic throughout the assay.

Many factors affect the cytotoxicity potential of AgNPs, such as their size, shape, surface chemistry, and cell type. However, in general, the cytotoxic effects are due to the oxidative stress caused by AgNPs and the release of Ag^+^. When internalized in cells, they can induce a series of effects, such as impairment of cell membrane, inflammatory response, DNA damage and genotoxicity, chromosome aberration, and apoptosis [103]. However, AgGO biosynthesized using flower extract of *Legistromia sepiosa* on a human embryonic kidney cell line (HEK-293) were non-toxic at an effective antibacterial concentration (94 µg/mL) [104]. Chemically synthesized AgNPs using NaBH_4_ with particle sizes lower than 20 nm were also reported to be safe at the highest tested concentration of 25 µg/mL as a topical agent when evaluated on HDFs and human epithelial keratinocytes for 24 h [105]. Although AgNPs are generally known for their toxic effects on human cells, they are safe when used at lower doses. In the current study, AgNPs were biologically synthesized using WETMM, thus avoiding the employment of toxic and harmful chemicals that may have lingered on the formed AgNPs. Additionally, AgNPs were coated with CS as a stabilizer, which could have also improved its overall safety profile by preventing any direct effects of AgNPs on the cells. 

Currently, no study has evaluated the cytotoxic effects of LTF on human skin cells. Still, when used in combination with AgNPs, it did not seem to significantly enhance its cytotoxicity. A study by Abdalla et al. [27] showed that AgLTF at concentrations (32, 62, and 125 µg/mL; 1:1 AgNPs to LTF ratio) was non-cytotoxic on human dermal fibroblasts (HDFs) for 48 h. However, cell viability determination using the AB test at 72 h showed a significant reduction in cell viability %, which may pose an issue in treatments requiring a longer duration. In a separate study, biosynthesized AgLTF using *Pleurotus ostreatus* (16–125 µg/mL) also showed characteristic dose and time-dependent cytotoxicity on HDFs over 72 h but were generally safe except for dose 125 µg/mL [54]. Although these reports exhibited the alarming cytotoxicity nature of AgLTF on HDFs at 125 µg/mL, it is essential to note that both studies used the extracted solution of AgLTF, which means that AgLTF was in direct contact with the cell cultures throughout the assay to exert its toxicity. This study formulated AgLTF-DsiRNA in hydrogel and further crosslinked it with genipin to allow a steady release of its active components over 24 h for SL and 72 h for DL, respectively, preventing the tested HaCaT from suffering from full-blown toxicity of AgLTF.

### 3.11. Cell Migration

Wound healing requires the complex coordination of various cell types, including keratinocytes, fibroblasts, endothelial cells, macrophages, and platelets [106]. It mainly involves cell proliferation and migration. In particular, the migration of keratinocytes is essential for wound re-epithelialization associated with angiogenesis, and any defects in this process may hamper wound closure [102]. Two methods are generally used to evaluate the cell migration of HaCaT, namely wound scratch assay and transwell cell migration assay. The wound scratch assay is the most common in vitro method to test compounds for their migratory properties due to its simplicity and cost-effective procedure [107]. Additionally, the scratch assay is comparable to the in vivo wound healing process because it involves the removal of cells using a pipette tip, which wounds the monolayer and causes destroyed and activated cells [108]. 

In a comprehensive study done by Ueck et al. (2016) to validate the in vitro scratch assay as a replacement for the expensive in vivo wound healing assay using pig model systems, they discovered that supplemented media are important to allow hyperglycemic conditions to affect in vitro wound healing of keratinocytes, and preincubation of the model in high glucose medium for at least 48 h is necessary to enable the glucose to execute its effects before testing [108]. In addition, HaCaT cells have strong cell-to-cell interaction and are facilitated by Ca^2+^-dependent interactions at the desmosome junction. Hence, prior to the scratch, washing with PBS is an essential step to weaken the cell-to-cell adhesion of HaCaT for easy removal, eliminating one of the disadvantages of performing manual scratches, as the cells tend to accumulate across the edge of the scratch due to this strong interaction [107]. 

**Figure 12:**
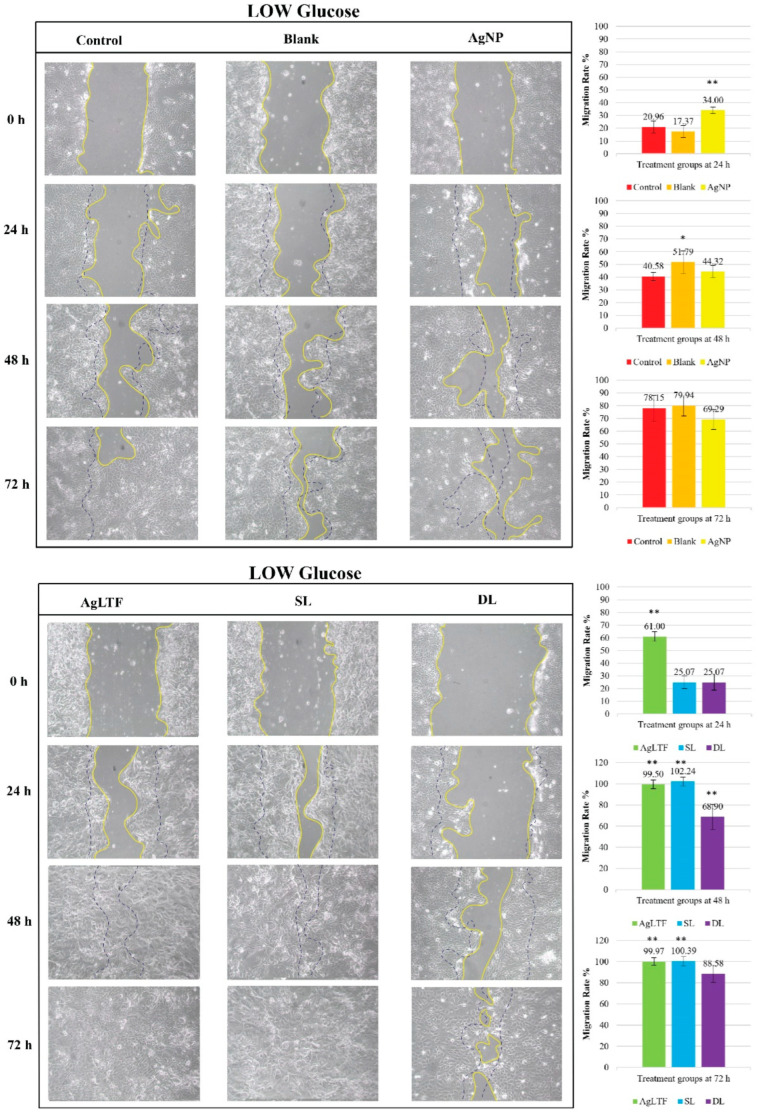

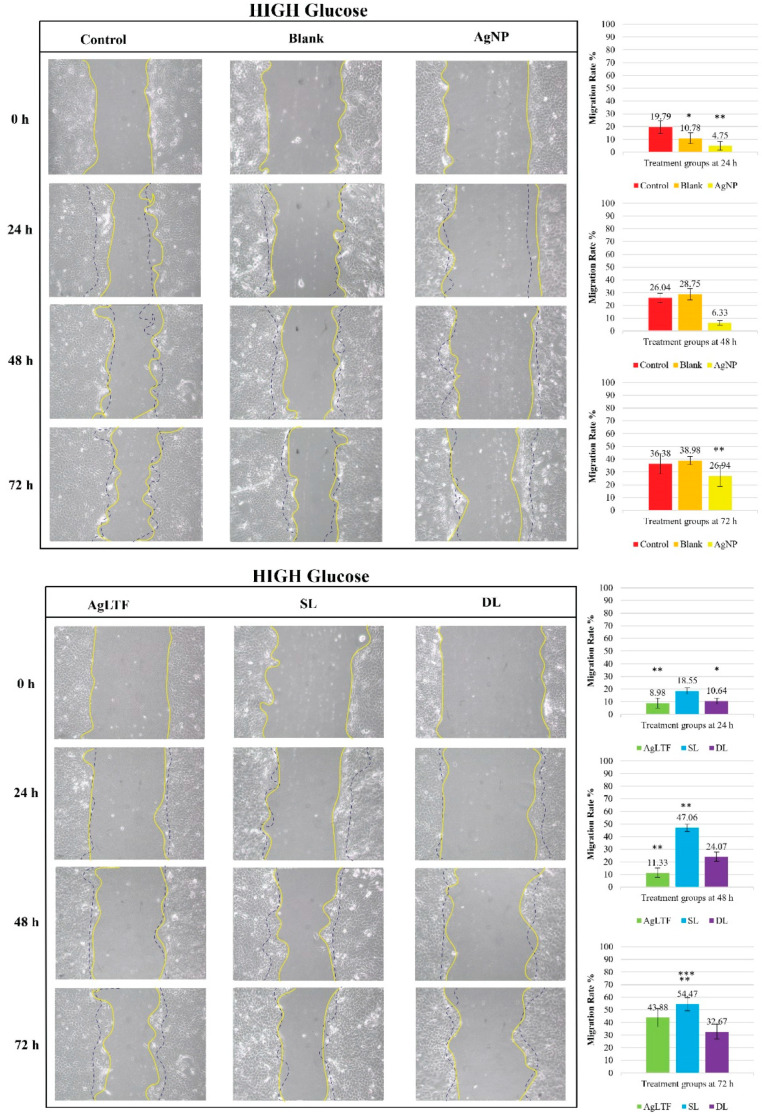
Wound scratch assay for control, blank, AgNPs, AgLTF, SL, and DL gels at time points 24, 48, and 72 h, *n* = 9. * statistically significant difference from the positive control (*p* < 0.05); ** statistically significant difference from the positive control (*p* < 0.01); *** statistically significant difference from AgLTF.

The in-house scratch assay confirmed that hyperglycemic conditions impaired the wound healing ability (Figure 12) when two controls were compared in different treatment groups. The control in the treatment group cultured in low glucose DMEM (78.15%) had a significantly higher migration rate than the control in the treatment group cultured in high glucose DMEM (36.38%) at 72 h (*p* < 0.01).

LTF and its effect on cell migration and proliferation are still being determined because many contradictory reports exist [109,110]. The AgLTF complex (32 and 62 µg/mL; 1:1 ratio of AgNPs and LTF) did not differ from the control group when tested on HDFs over 72 h [27]. Furthermore, bovine- and human-derived LTF also had distinct effects against MAC-E (bovine mammary epithelial cell line) and MCF-7 (human breast tumor epithelial cell line). Bovine-derived LTF had inhibitory effects on the cell proliferation of MAC-E cells and did not affect MCF-7 cells. In contrast, human-derived LTF had a slight inhibitory effect on MCF-7 cells and no effect on MAC-E cells [111]. However, various forms of LTF, including recombinant LTF, have shown positive cell proliferation and migration results at concentrations of 10–1000 µg/mL in multiple studies [109,110,112]. 

In a study, LTF with an iron saturation level of over 90% had the highest proliferation activity (3.5×) greater than other forms of LTF with lower iron saturation levels when tested on four cell lines [110]. LTF is also reportedly able to stimulate HDF migration at 50–200 µg/mL in a dose-dependent manner [109] and human keratinocytes at 10–100 µg/mL [112]. The mechanism by which LTF stimulates the proliferation and migration of cells is unclear. Still, this is probably because it can bind to lipoprotein receptor-related protein 1 (LRP-1), a major LTF receptor in mammalian cells, to stimulate growth. This LRP-1 receptor can be found mainly in keratinocytes and fibroblasts [112]. In addition, LTF also has synergistic effects with other growth factors and can delay cell apoptosis [110]. In the current study, when HaCaT cells were cultured in low glucose DMEM, the migration rate of cells treated with AgLTF was significantly higher than the control group from 24 h of incubation until the end of the assay at 72 h. AgLTF achieved a 100% wound closure rate at 48 h, surpassing the migration rate of the control group even at 72 h. AgNPs alone only reached 69.29% at 72 h, which is a testament to LTF’s pro-migratory effects on skin cells. However, in the high glucose DMEM treatment group, AgLTF (43.88%) achieved only a comparable migration rate to the control group (36.38%). Considering that the migration rate of AgNPs alone was significantly lower than the control at 72 h, incorporating LTF into AgNPs resulted in an improved overall migration rate of the complex, albeit it was non-significant to control.

As for the SL gels containing DsiRNA, they were only able to achieve higher migration rates of HaCaT cells (54.47%) than the control group (36.38%) after 48 h when cultured in high glucose DMEM (*p* < 0.01). At 72 h, SL had a significantly higher migration rate than both the control and AgLTF groups in the high glucose treatment groups. Generally, incorporating DsiRNA into the formulation was derived from a study by Syeda et al. (2012), which concluded that diabetic wounds are difficult to treat due to the increased PGT protein expression and mRNA levels. It negatively affects PGE_2_ and VEGF levels, wound closure, and angiogenesis. In this study, the sequence of the DsiRNA was designed to target a specific mRNA for PGT and cause degradation at the cellular level. Hence, this DsiRNA silences the protein-coding genes and restores the PGE_2_ to a normal level. Additionally, DL gel was included as a treatment sample to test and validate the hypothesis that treating wounds in their respective phases (inflammation phase: 1–3 days and proliferation phase: 2–10 days) is better than focusing on a single healing stage [51]. However, DL achieved underwhelming results for both the low glucose treatment group (88.58%) and the high glucose treatment groups (32.67%) at 72 h, which were non-significant to the control group, probably due to insufficiency in the study duration. Ma et al. (2020) demonstrated a 5-day release of a conditioned medium of RAW 247 cells in sodium alginate microparticles to stimulate the formation of vascularized granulation tissue during the proliferation stage in an 18-day in vivo wound healing study. In this study, only 72 h of wound scratch assay duration was permitted due to the potential toxicity of AgNPs. As previously mentioned, the proliferation phase in wound healing usually occurs after the inflammation phase on day 2 up to day 10 after injury. Therefore, there is a high chance that the synchronized effect of the biological proliferation of cells and DsiRNA did not occur. Hence, a simple SL gel remains superior to a DL gel in this research setting. Moreover, further research involving prolonged-release DsiRNA on at least a 5-day wound scratch assay is warranted to investigate whether the migratory capacity of HaCaT cells is enhanced.

**Figure 13:**
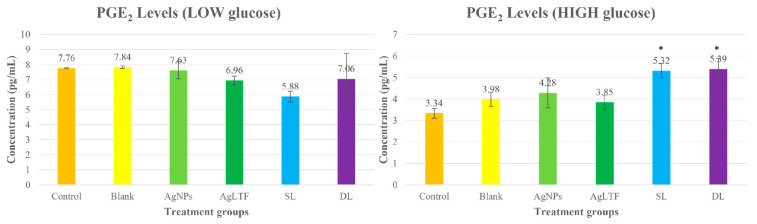
Measurements of PGE_2_ of the control, blank, AgNPs, AgLTF, SL, and DL in high and low glucose culture medium using the ELISA technique, *n* = 5. * statistically significant different from the positive control of their respective culture medium groups (*p* < 0.05).

The findings from the scratch assay were further supported by the protein levels of PGE_2_ obtained using the ELISA technique (Figure 13). PGE_2_ is vital for wound healing because it stimulates angiogenesis via the VEGF protein [37]. Therefore, when PGE_2_ increases, angiogenesis and the wound healing rate are also increased. PGE_2_ levels in the low glucose treatment group were much higher than the PGE_2_ levels in the high glucose treatment group (*p* < 0.01). Despite this, PGE_2_ levels of SL and DL, when cultured in high glucose DMEM, were significantly higher than their control groups, implicating the importance of DsiRNA to correct PGT overexpression in a hyperglycemic condition. Overall, the treatment results of SL and DL gel were not comparable to those in the treatment groups cultured in low glucose DMEM. This reflects a normal person’s wound healing capacity, probably because of other cellular impairments in diabetic wounds. For instance, the basic fibroblast growth factor (bFGF) signaling protein Rac1, which is essential to promote cell migration and increase fibronectin expression, is abnormally activated in hyperglycemic conditions to delay wound healing [106]. Since DsiRNA in this study was only used to correct the overexpression of PGT proteins in diabetic wounds, other cellular abnormalities may persist and delay wound healing to an extent. Ultimately, LTF and DsiRNA complexed to AgNPs in hydrogel enhanced cell migration of HaCaT cells and improved the wound healing capacity of diabetic wounds, although it was not to its full capacity.

## 4. Conclusions

In this study, AgNPs were successfully complexed with LTF to form AgLTF, and the fabricated complex was in crystalline nano-sized particles and primarily spherical. AgLTF demonstrated antibacterial activity against *S. aureus*, *E. coli,* and *P. aeruginosa*. AgLTFs were later successfully loaded with DsiRNA and packaged in SL and DL hydrogels made of gelatin and genipin with two different drug release profiles. DL hydrogels demonstrated slower release for AgNPs, LTF, and DsiRNA than SL hydrogels at 72 h. Antibacterial tests for SL and DL hydrogels exhibited positive effects against Gram-positive and Gram-negative bacteria, consistent with the antibacterial results obtained in its solution form. AgLTF, SL, and DL hydrogels showed higher biofilm inhibition against *P. aeruginosa* and *S. aureus* than the control group, indicating that LTF plays an essential role in combating biofilm-forming pathogens by enhancing the anti-biofilm effect of AgNPs. AgLTF-DsiRNA in SL gel containing 125 µg/mL AgNPs, 250 µg/mL LTF, and 0.015 µg/mL DsiRNA were non-cytotoxic at 72 h treatment with HaCaT cells. As for the cell migratory properties, SL and AgLTF gels increased the migration rate of HaCaT cells in both low and high glucose DMEM, indicating an improved wound healing rate due to the pro-migratory effects of DsiRNA and LTF. Moreover, PGE_2_ protein levels were also increased in SL hydrogels, showing that PGE_2_ was indeed underexpressed in hyperglycemic conditions and was improved via the effects of DsiRNA to enhance wound healing rate in diabetic patients. However, DL hydrogels demonstrated underwhelming results regarding their migratory capacity, probably due to the slow release of DsiRNA from the hydrogels. On this basis, further optimization of AgLTF is needed to improve its biocompatibility so that the in vitro wound healing assay can be extended to at least 5 days. This is to synchronize the migratory effects of DsiRNA with the biological proliferation phase in the wound healing process, which occurs from day 2 to day 10, and to observe the additional impact of the prolonged release of DsiRNA on diabetic wound healing. Perhaps in a future study, a diabetic wound model using rats or mice could also be considered to solidify the in vitro results obtained from this study. Nevertheless, these findings provide further understanding and knowledge on the formation of multipronged AgNPs consisting of DsiRNA and LTF as a potential application of chronic wound therapy.

## Figures and Tables

**Table 1:** The mean particle size, PDI, and zeta potential of AgNPs, AgLTF, and AgLTF-DsiRNA, *n* = 3.

	Particle Size (nm)	PDI	Zeta Potential (mV)
AgNPs	58.40 ± 6.30	0.52 ± 0.03	+31.7 ± 4.8
AgLTF	113.32 ± 24.98	0.23 ± 0.014	+18.26 ± 1.53
AgLTF-DsiRNA	157.40 ± 5.00	0.35 ± 0.013	+16.3 ± 1.23

**Table 2:** EE of AgNP-DsiRNA and AgLTF-DsiRNA at various concentrations, *n* = 4.

Concentration of AgNPs (µg/mL)-DsiRNA (ng/mL)	EE (%)	Concentrations of AgLTF (µg/mL)-DsiRNA (ng/mL)	EE (%)
125/15	69.51 ± 2.4	125/15	71.7 ± 0.47
500/15	68.35 ± 0.15	500/15	68.19 ± 0.31

**Table 3:** Physical characteristics of crosslinked gelatin (3% *w*/*v*) hydrogel.

Concentration of Genipin in the Upper Layer (% *w*/*v*)	Appearance	Texture	Smoothness	Stickiness
0.005	Clear & homogenous	Slightly bouncy when touched	Smooth	Intermediate
0.01	Clear & homogenous	Rigid	Smooth	High
0.02	Clear & homogenous	Rigid	Smooth	High
0.04	Clear & homogenous	Rigid	Smooth	High

**Table 4:** Zone of inhibition for *S. aureus*, *P. aeruginosa,* and *E. coli* for the AgLTF, SL, and DL gels, *n* = 3.

Samples	Zone of Inhibition (mm)		
	*S. aureus*	*P. aeruginosa*	*E. coli*
Ciprofloxacin (20 µg/mL)	31.7 ± 2.9	23 ± 3.6	36.3 ± 1.7
CPX disk (5 µg)	33.0 ± 1.0	33.7 ± 1.2	33.0 ± 1.0
LTF (125, 250, 500 & 1000 µg/mL)	-	-	-
AgLTF (125/250 µg/mL)	10.3 ± 1.5	7.3 ± 0.6	11.3 ± 7.5
AgLTF (1000/2000 µg/mL)	11.3 ± 1.2	9.3 ± 0.6	13.3 ± 2.9
SL gel containing AgLTF (125/250 µg/mL)	10.3 ± 1.5	13.3 ± 0.6	13.3 ± 0.6
DL gel containing AgLTF (125/250 µg/mL)	10.0 ± 1.7	14.7 ± 0.6	15.0 ± 1.0

## Data Availability

The data presented in the study is available in the article.
